# New Steroids Obtained from *Ailanthus altissima* Leaves Inhibit the Invasive Bacteria *Xanthomonas oryzae* pv. *oryzae* and *Pseudomonas syringae* pv. *maculicola*

**DOI:** 10.3390/molecules30122576

**Published:** 2025-06-13

**Authors:** Yuhong Yang, Yue Wu, Zhengyi Gao, Zhixiang Liu, Juan Hua, Shihong Luo

**Affiliations:** Engineering Research Center of Protection and Utilization of Plant Resources, College of Bioscience and Biotechnology, Shenyang Agricultural University, Shenyang 110866, China; 2001500032@syau.edu.cn (Y.Y.); 16697789978@163.com (Z.G.); liuzhixiang327@163.com (Z.L.); huajuan@syau.edu.cn (J.H.)

**Keywords:** invasive bacteria, *Ailanthus altissima*, steroids, *Xanthomonas oryzae* pv. *oryzae*, *Pseudomonas syringae* pv. *maculicola* ES4326, antibacterial activity

## Abstract

Invasive bacteria have caused tremendous losses to global ecosystems and agricultural production, yet effective control measures remain elusive. Plant specialized metabolites are being investigated as an important source of antimicrobial active substances. And *Ailanthus altissima* is an abundant tree widespread throughout Northeast China. In this study, we identified 21 compounds from *A. altissima* leaves, including steroids, terpenes, phenolics, and coumarins. Two new steroidal compounds, ailanstigol A (**1**) and ailanstigol B (**2**), and one new coumarin (2′*R*,3′*R*)-7-(2′,3′,6′-trihydroxy-3′-methylhexyloxy)-6,8-dimethoxycoumarin (**3**) were isolated. Antibacterial screening revealed that compounds **1** and **2** exhibited inhibitory activity against two invasive bacteria, *Xanthomonas oryzae* pv. *oryzae* PXO 71A and PXO 86A and *Pseudomonas syringae* pv. *maculicola* ES4326. Further mechanistic screening unveiled that the steroidal compounds **1** and **2** may inhibit bacterial growth and reproduction by reducing cell viability, disrupting the cell membrane and increasing protein leakage, and inhibiting biofilm formation. In summary, our results enriched the known chemical diversity of *A. altissima* and provided a foundation for investigating the mechanisms by which steroidal compounds inhibit invasive bacterial growth.

## 1. Introduction

Invasive species are posing an increasing global risk [1], and their invasion and spread have caused tremendous losses to global ecosystems and the economy [2]. Invasive bacteria are introduced into new areas through the introduction of other invasive species or through human activities, and where they are pathogenic, these bacteria can pose serious threats to local plants, including crops [3]. For example, the invasive bacterium *Pseudomonas syringae* pv. *maculicola* can cause leaf spot in a variety of Brassicaceae plants, including cabbage, cauliflower, broccoli, Chinese cabbage, radish, and turnip, and has posed a serious threat to the cultivation of Brassicaceae worldwide [4]. The bacterium *P. syringae* pv. *actinidiae* can lead to kiwifruit bacterial canker and has caused enormous damage to the economy of New Zealand [5]. One of the most severe bacterial diseases in rice crops in China is rice leaf blight, which is caused by the Gram-negative bacterium *Xanthomonas oryzae* pv. *oryzae*. Rice leaf blight outbreaks are common in China and typically result in yield reductions of between 20 and 30%, with severe cases resulting in reductions of up to 50% or even total crop failure [6]. Invasive bacteria typically exhibit rapid growth and strong reproductive capacity, and native plants typically have difficulty defending against them. The prevention and control of invasive bacteria has become an important topic in plant disease research.

Before the “era of synthesis”, 80% of medicines, fungicides, and pesticides were derived from the roots, bark, or leaves of plants [7]. Plant-derived bioactive compounds can exhibit excellent antimicrobial effects, with therapeutic efficacy as strong as that of synthetic chemical agents. Additionally, they are abundant, environmentally friendly, and do not cause environmental pollution or residual toxicity issues, meaning that the study and use of these compounds has a broad and promising future. There are many excellent examples of antimicrobial compounds isolated from plants. For example, glycoalkaloids and glucosinolates extracted from *Solanum nigrum* and *Armoracia rusticana* exhibit inhibitory activity against the Gram-positive bacteria *Bacillus thuringiensis* and *B. amyloliquefaciens* [8]. Naringin and quercetin show significant antibacterial activity against *B. subtilis*, *Escherichia coli*, and *Staphylococcus aureus*, while flavonoids in chestnuts are able to eradicate spoilage bacteria, including *Megasphaera* spp. and *Proteus* spp. [9]. Polyphenols from moss species and cloves (*Syzygium aromaticum*) exhibit antibacterial activity against *E. coli*, *Proteus mirabilis*, *Klebsiella pneumoniae*, *Enterobacter cloacae*, and *Salmonella* spp. [10].

Compared with synthetic chemical compounds, plant-derived bioactive compounds typically show lower toxicity and fewer side effects. Moreover, most of these compounds have minimal influence on the environment, aligning with the goal of sustainable development. The antimicrobial activity of plant-derived bioactive compounds therefore provides an abundant and attractive natural resource for the development of new antimicrobial agents. This is reflected in the market, with the annual growth rate of the traditional agricultural chemical market being currently 5.5%, while that of the biopesticide sector is growing at a rate of 16% per year.

*Ailanthus altissima* (Mill.) Swingle, a deciduous tree belonging to the family of Simaroubaceae, is widely distributed in tropical and warm regions, and it is abundant in Northeast China [11]. The species has become an invasive alien plant in Europe and North America. The aqueous extracts of *A. altissima* leaves and roots are used in Northeast China as a traditional form of pest control, including the control of *Pieris rapae*, *Helicoverpa armigera*, and aphids [12]. *A. altissima* is rich in chemical compounds, and many types of specialized metabolites, including alkaloids, terpenoids, steroids, and flavonoids, have been isolated from the plant. These specialized metabolites also show diverse biological activities; for example, quassinoid glycosides derived from *A. altissima* inhibit the multiplication of tobacco mosaic virus [13], and ailanthone, a quassinoid compound, strongly inhibits the expression of RSV protein–coding genes, thereby reducing the damage caused by rice stripe disease [14]. However, although many diverse compounds are known from *A. altissima* [15], their efficacy against invasive pathogenic bacteria and the mechanisms of any potential interactions remain unknown.

## 2. Results and Discussion

### 2.1. Isolation and Identification of Compounds from the Leaves of Ailanthus Altissima

**Figure 1:**
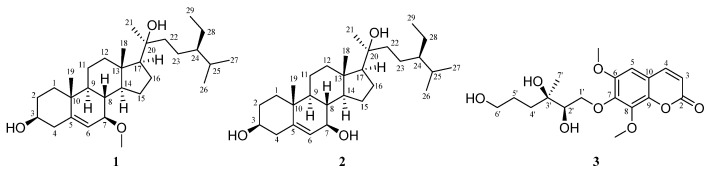
Chemical structures of new compounds from the leaves of *Ailanthus altissima*.

Using phytochemical methods, 21 compounds were isolated and identified from the ethyl acetate extract of *A. altissima* leaves (Figure 1). These compounds included steroids (compound **4**), phenols (compounds **5**–**17**), terpenoids (compounds **17**–**19**), coumarins (compound **20**), and alkaloids (compound **21**). The compounds were identified as stigmast-5-ene-3*β*,7α,20*ξ*-triol (compound **4**) [16], cinnamic acid (compound **5**) [17], benzoic acid (compound **6**) [18], (E)-3-(4-hydroxyphenyl)acrylic acid (compound **7**) [19], 4-hydroxybenzoic acid (compound **8**) [20], methyl gallate (compound **9**) [21], (Z)-3-(4-hydroxyphenyl) acrylic acid (compound **10**) [19], methyl caffeate (compound **11**) [22], 4-hydroxy-3-methoxybenzoic acid (compound **12**) [23], caffeic acid (compound **13**) [24], methyl-3,4,5-trihydroxybenzoate (compound **14**) [25], luteolin (compound **15**) [26], quercetin (compound **16**) [27], (-)-loliolide (compound **17**) [28], (+)-dehydrovomifoliol (compound **18**) [29], (3S,5R,6S,7E)-3,5,6-trihydroxy-7-megastigmen-9-one (compound **19**) [29], scopoletin (compound **20**) [30], and indazole (compound **21**) [31] (Figure S1).

### 2.2. The Structural Elucidation of New Compounds

**Figure 2:**
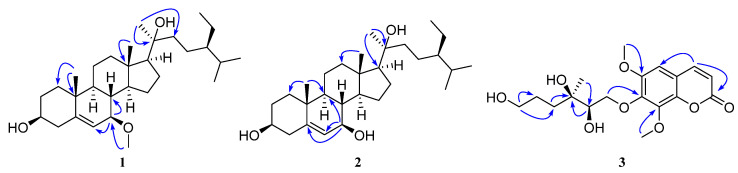
The main HMBC (H→C) correlation of compounds **1**–**3**.

**Figure 3:**
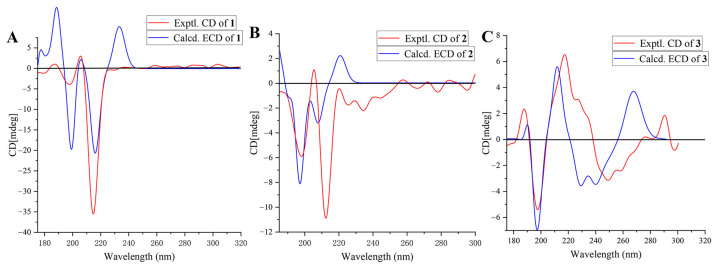
Comparison of experimental circular dichroism (CD) and calculated electronic circular dichroism (ECD) spectra of compounds **1**–**3**. (**A**) experimental CD and calculated ECD spectra of compound **1**; (**B**) experimental CD and calculated ECD spectra of compound **2**; (**C**) experimental CD and calculated ECD spectra of compound **3**.

Compound **1** is a white solid, and it exists in 75% ethanol, methanol, acetone, and dichloromethane extracts of *A. altissima* leaves (Figure 1 and Figure S29). Based on HR-ESI-MS (*m*/*z*_obsd_ 483.3816 [M + Na]^+^; *m*/*z*_calcd_ 483.3814), its molecular formula was determined to be C_30_H_52_O_3_ with 5 degrees of unsaturation. The low field of the ^1^H NMR spectrum (Table 1) showed three singlet methyl groups at *δ*_H_ 0.85 (3H, s), *δ*_H_ 0.99 (3H, s), and *δ*_H_ 1.24 (3H, s); two doublet methyl groups at *δ*_H_ 0.86 (3H, d, *J* = 6.5 Hz) and *δ*_H_ 0.87 (3H, d, *J* = 6.5 Hz); and one triplet methyl group at *δ*_H_ 0.89 (3H, t, *J* = 7.3 Hz). In the midfield, there were two oxygenated methylene protons at *δ*_H_ 3.34 (br s) and *δ*_H_ 3.45 (m), and one methoxy proton at *δ*_H_ 3.35 (3H, s). In the high field, there was one double singlet proton at *δ*_H_ 5.77 (d, *J* = 4.1 Hz), with the remaining proton signals ranging from *δ*_H_ 0.94 to *δ*_H_ 2.29 (Table 1). ^13^C NMR and DEPT spectra indicated that the compound contained 30 carbon atoms, including 7 methyl groups, 10 methylene groups, 9 methyne groups (comprising 2 oxidized methine carbons *δ*_C_ 72.2 and 75.2 and 1 methylene carbon *δ*_C_ 121.5), and 4 quaternary carbons. Based on 1D and 2D NMR spectra, we inferred that compound **1** was a stigmasterol steroid. The skeletal carbon signals of **1** were similar to those of compound **4** (stigmast-5-ene-3*β*,7*α*,20*ξ*-triol) [16,32]. The difference between these two compounds is that one of the oxygenated methine carbons (*δ*_C_ 75.2) in compound **1** appears at a lower field position in the ^13^C NMR spectrum compared to the corresponding carbon (*δ*_C_ 65.2) in **4**. From the HMBC spectrum of compound **1** (Figure 2), we found that H-6 (*δ*_H_ 5.77) and the methoxy proton signal *δ*_H_ 3.35 correlated with the oxygenated methine carbon *δ*_C_ 75.2, indicating that C-7 of compound **1** was methoxylated. Analysis of ROESY spectra revealed that H-7 correlated with H-9 and H-14, indicating that the methoxy group at the C-7 position had *β*-configuration. And the calculated ECD curve was consistent with the experimental CD curve of 7*R*, 17*S*, indicating that the absolute configuration of compound **1** was 7*R*, 17*S* (Figure 3). In this way, the structure of **1** was eventually determined and was named ailanstigol A.

Compound **2** is also a white solid, and it will not be transformed into compound **1** in methanol solvent (Figure S30). In HR-ESI-MS, a molecular ion peak was observed (*m*/*z*_obsd_ 469.3644 [M + Na]^+^; *m*/*z*_calcd_ 469.3658) that suggested a molecular formula of C_29_H_50_O_3_ with 5 degrees of unsaturation. Based on 1D and 2D NMR spectra, we inferred that **2** was a steroid with a stigmasterol skeleton, which is similar to the structure of compound **1**. Analysis of ^13^C NMR and DEPT spectra revealed that **2** lacked only one methoxy carbon compared to **1**, suggesting that the 7-OMe group was absent in **2**. Further analysis of HMBC and ^1^H-^1^H COSY spectra revealed that C-7 is replaced by a hydroxyl group in **2** (Figure 2). The ROESY spectra revealed that H-7 (*δ*_H_ 3.72) was correlated with H-9 (*δ*_H_ 1.02) and H-14 (*δ*_H_ 1.13), which means that the H-7 of compound **2** also showed an α configuration. Although a hydroxyl group with a β configuration of 7-OH has been reported from stigmast-5-ene-3*β*,7*α*,20*ξ*-triol [16], the chemical shift value at the C-7 in compound **2** was *δ*_C_ 73.7, while the values at the C-7 in the two reported compounds were *δ*_C_ 65.3 [32] and 65.2 [16]. And the calculated ECD curve of the 7*R*, 17*S* was close to the experimental CD curve of compound **2** (Figure 3); in this way, the structure of **2** was eventually determined, and the compound was named ailanstigol B.

Compound **3** is a white powder. In the HR-ESI-MS analysis, a molecular ion peak was observed (*m*/*z*_obsd_ 391.1371 [M + Na]^+^; *m*/*z*_calcd_ 391.1369) that suggested a molecular formula of C_18_H_24_O_8_ with 6 degrees of unsaturation. The ^1^H NMR spectrum (Table 2) showed three singlet methyl groups (*δ*_H_ 1.13, 3.90, and 4.00) and three olefinic hydrogen protons (*δ*_H_ 6.34, 7.10, and 7.92) (Table 2). ^13^C NMR and DEPT spectra indicated that the compound contains 18 carbons, which were classified as three methyl groups (including two oxidized methyls at *δ*_C_ 56.7 and 62.0), four methylene groups (including two oxidized methylenes at *δ*_C_ 63.2 and 77.0), four methine groups (including one oxidized methane at *δ*_C_ 76.0), and seven quaternary carbons (including one ester carbonyl carbon at *δ*_C_ 160.4). From the 1D and 2D NMR spectra, we speculated that compound **3** could be a coumarin with a benzopyran-α-pyrone core structure. The skeletal carbon signals of **3** are very similar to the known compound (2′*R*,3′*R*)-7-(2′,3′-dihydroxy-3′,7′-dimethylocta-6′-enyloxy)-6,8-dimethoxycoumarin [33], with the difference between these two being that **3** lacks two methyl groups and one double bond. Based on ^13^C NMR and HMBC data, we found that the C-6′ of compound **3** was an oxidized methylene group (*δ*_C_ 63.2, *δ*_H_ 3.53) (Figure 2), indicating that the 6′-OH has replaced the original double bond and two methyl substituents. And the calculated ECD curve was consistent with the experimental CD curve of 2′*R*, 3′*R*, indicating that the absolute configuration of compound **3** was 2′*R*, 3′*R* (Figure 3). In this way, the structure of **3** was eventually determined, and the compound was named (2′*R*,3′*R*)-7-(2′,3′,6′-trihydroxy-3′-methylhexyloxy)-6,8-dimethoxycoumarin (Figure 1).

### 2.3. Growth Inhibition Activity Screening of Compounds Against X. oryzae pv. oryzae PXO 71A and PXO 86A and P. syringae pv. maculicola ES4326

**Figure 4:**
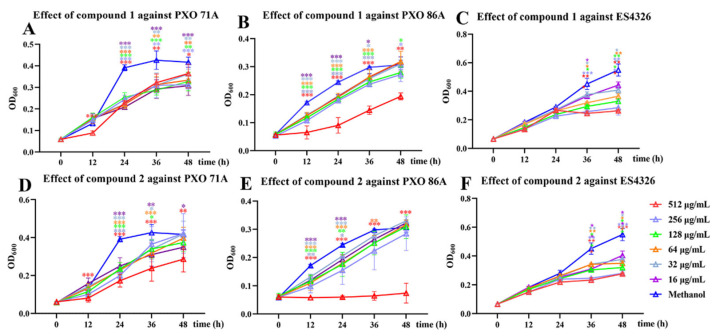
The growth inhibitory activity of compounds **1** and **2** against agricultural invasive bacteria. (**A**–**C**) represent the results of the inhibitory activity screening of compound **1** against the bacteria *Xanthomonas oryzae* pv. *oryzae* PXO 71A, PXO 86A, and *Pseudomonas syringae* pv*. maculicola* ES4326. (**D**–**F**) represent the results of the inhibitory activity screening of compound **2** against the bacteria PXO 71A, PXO 86A, and ES4326. An independent samples *t*-test was used to compare the experimental and control groups, where *p* < 0.05 was “*”, *p* < 0.01 was “**”, and *p* < 0.001 was “***”.

Using the two-fold dilution method, the growth inhibitory activities of compounds **1** and **2** against *X. oryzae* pv. *oryzae* PXO 71A and PXO 86A and *P. syringae* pv*. maculicola* ES4326 were screened. The results showed that, at a concentration of 512 µg/mL, compound **1** significantly inhibited the growth of PXO 71A and PXO 86A strains, with the highest inhibition rates observed being 52.62 ± 2.70% and 62.93 ± 11.32%, respectively, following 24 h of treatment. The IC_50_ values were found to be 485.85 ± 28.26 and 314.16 ± 36.76 µg/mL for PXO 71A and PXO 86A strains, respectively (Figure 4A,B). After the ES4326 strain was cultured in compound **1**, the growth of the strain was found to be inhibited from 24 h to 48 h, with the inhibition rate reaching a peak of 52.05 ± 1.37% at 48 h at 512 µg/mL of compound **1** (Figure 4).

We found that compound **2** exhibited significant growth inhibitory activity against PXO 71A and PXO 86A strains. And as the concentration increases, the inhibitory effects also become more pronounced. When the PXO 71A or PXO 86 strain was cultured for 24 h with compound **2** at a concentration of 512 μg/mL, the inhibition rates were 55.83 ± 8.60% or 75.70 ± 2.29%, respectively (Figure 4D,E). The IC_50_ values of compound **2** against PXO 71A and PXO 86A strains were 354.09 ± 23.75 and 274.12 ± 67.19 μg/mL, respectively. Meanwhile, compound **2** also exhibited a certain inhibitory effect on the growth of the ES4326 strains. When the ES4326 strain was treated with 256 μg/mL of compound **2** for 48 h, the inhibition rate was found to be 49.04 ± 3.06% (Figure 4F). The inhibition rate of 4 μg/mL kanamycin (positive control) against the PXO 71A strain after co-culturing for 24 h was 85.67 ± 3.25%. The PXO 86A strain was treated with streptomycin (positive control) for 24 h with an IC_50_ of 21.15 ± 2.38 μg/mL, and the inhibition rate of 4 μg/mL kanamycin against the ES4326 strain after treatment for 24 h was 82.27 ± 3.25% (Figure S31).

Meanwhile, the double dilution method was used to screen compounds **13**, **15**, and **17** for antimicrobial activity against the PXO 71A, PXO 86A, and ES4326 strains. Compound **13** exhibited a weak inhibitory effect against the PXO 71A, PXO 86A, and ES4326 strains. Compound **15** had almost no effect on the growth of the ES4326 strain but exhibited weak inhibitory effects against the PXO 71A and PXO 86A strains. Culture of PXO 71A and PXO 86A for 36 h with 512 μg/mL of compound **15** resulted in inhibition rates of 22.38 ± 2.67% and 31.22 ± 2.29%, respectively. However, compound **17** only exhibited a weak inhibitory effect against PXO 86A (28.24 ± 2.71% after treatment with compound **17** at 256 μg/mL for 24 h (Figure S2).

*A. altissima* exhibits remarkable biological activities and is recognized as a versatile tree. For example, its leaves possess antibacterial properties, its bark demonstrates neuroprotective effects, and its seeds and branches exhibit notable anti-inflammatory activity [34]. According to previous studies, the leaves of *A. altissima* are abundant in polyphenols, sesquiterpenes, and triterpenoids, among which the phenolic composition of leaf extracts exhibits excellent antibacterial activity against *Escherichia coli* and *Candida albicans* [35]. In addition, the triterpenoid compound ailanthone extracted from *A. altissima* leaves can significantly inhibit the growth of garden cress and radish when its concentration is 7.5 mg/L, and the inhibition rate can reach up to 95% after 30 days of treatment [36]. In this study, two new steroidal compounds, ailanstigol A (**1**) and ailanstigol B (**2**), a new coumarin (2′*R*,3′*R*)-7-(2′,3′,6′-trihydroxy-3′-methylhexyloxy)-6,8-dimethoxycoumarin (**3**), and 18 known compounds were isolated from *A. altissima* leaves. And the side chain of compound **3** may be derived from the ten-carbon chains provided by GPP as precursors, and the terminal methyl groups were further oxidized to form the eight-carbon side chain [37]. The identification of these new compounds has enriched the chemical diversity of *A. altissima* leaves and laid a foundation for exploring their extensive biological activities.

In addition to phenolic compounds, steroid compounds also exhibit remarkable antibacterial activity; for example, azasteroids are known to inhibit the growth of *Bacillus subtilis* and *Escherichia coli* at concentrations of 5–10 mol/L [38], and the steroid *β*-stigmasterol, which was isolated from the stem of *Cola lateritia* K. Schum., exhibits inhibitory activity against several species of bacteria, including *Bacillus subtilis*, *Staphylococcus epidermidis*, *Enterococcus faecalis*, *Mycobacterium smegmatis*, and *S. aureus* [39]. Stigmast-4-ene-3,6-dione isolated from *Hedyotis pilulifera* exhibits significant antibacterial activity against *Mycobacterium smegmatis* and *Bacillus subtilis*, with an MIC value of 5 μg/mL [40]. In this experiment, we found that compounds **1** and **2** exhibit potent inhibitory activity against *X. oryzae* pv. *oryzae* PXO 71A, PXO 86A, and *P. syringae* pv. *maculicola* ES4326. At concentrations of 512 µg/mL, compound **2** exhibits the highest inhibitory activity against the PXO 86A strain, with a bacterial inhibition rate of 75.70 ± 2.29%, while **1** exhibits excellent inhibitory activity against the ES4326 strain, with an inhibition rate of 52.62 ± 2.70%. These results demonstrate that steroid compounds play a crucial role in the management of invasive bacteria *X. oryzae* pv. *oryzae* and *P. syringae* pv. *maculicola*. Moreover, compound **2** with a hydroxyl group at the C-7 site can exert better antibacterial effects against these two invasive bacteria than compound **1** with a methoxy group at the same site.

In future research guided by the principles of green agriculture, the inhibitory efficacy of steroid compounds against *X. oryzae* pv. *oryzae* and *P. syringae* pv. *maculicola* could potentially be enhanced through chemical modifications, such as the introduction of flexible alkyl chains or functional hydroxyl groups. Furthermore, the key synthesis process starting from acetyl coenzyme A, which is crucial for the large-scale production and application of these steroid compounds, is also the focus of future research.

### 2.4. The Ailanstigols A and B Lead to Intracellular Protein Leakage

**Figure 5:**
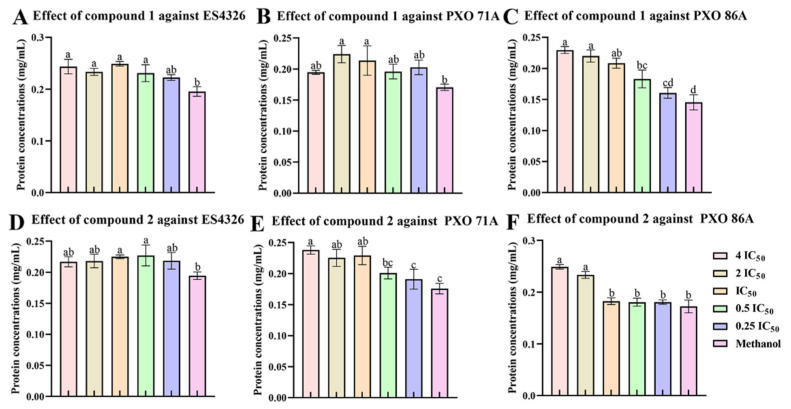
Determination of intracellular protein leakage after culture of *Xanthomonas oryzae* pv. *oryzae* PXO 71A, PXO 86A, and *Pseudomonas syringae* pv*. maculicola* ES4326 in the presence of compounds **1** or **2**. (**A**–**C**) The results of the measurement of intracellular protein leakage after treatment of the ES4326, PXO 71A, or PXO 86A strain with compound **1** for 6 h; (**D**–**F**) the results of the measurement of intracellular protein leakage after treatment of the ES4326, PXO 71A, or PXO 86A strain with compound **2** for 6 h. IC_50_: half maximal inhibitory concentration. The IC_50_ values of compound **1** against the PXO 71A, PXO 86A, and ES4326 strain were determined to be 485.85 μg/mL, 314.16 μg/mL, and 289.23 μg/mL, respectively. The IC_50_ values of compound **2** against the PXO 71A, PXO 86A, and ES4326 strains were determined to be 354.09 μg/mL, 274.12 μg/mL, and 476.18 μg/mL, respectively. One-way ANOVA and Tukey’s tests were used to compare mean differences, and different lowercase letters (a, b, c, and d) represent significant differences (*p <* 0.05).

Treatment of bacteria with certain compounds could disrupt the bacterial cell membrane, leading to the outflow of intracellular macromolecules such as nucleic acids and proteins, and ultimately resulting in cell death. In this experiment, the ES4326, PXO 71A, or PXO 86A strain was cultured in the presence of compound **1** or **2**. Cell supernatants were collected by centrifugation after culture, and the protein content was measured. The results showed that compound **1** significantly increased the leakage of intracellular proteins in the ES4326 and PXO 86 strains. Following 6 h of culture of ES4326 with compound **1** at 4 times the IC_50_ concentration, compared with the solvent control group, the protein leakage increased by 41.41 ± 11.27% (Figure 5A). Furthermore, compound **1** at 4 times the IC_50_ concentration increased the leakage of the PXO 86 strain protein by 44.43 ± 2.54% compared with the solvent control group, and there was a significant dose–dependent relationship between the compound treatment concentration and the amount of protein leakage in bacterial cells (Figure 5C). In contrast, compound **1** caused only very little intracellular protein leakage in the PXO 71A strain (Figure 5B).

Compound **2** also increased the leakage of intracellular proteins in the PXO 71A and PXO 86A strains, but the effect was weaker than that of compound **1**. Following 6 h of culture of the PXO 71A strain with compound **2** at 4 times the IC_50_ concentration, the protein leakage increased by 35.41 ± 3.82% compared with the solvent control group (Figure 5E). Indeed, at 2 or 4 times the IC_50_ concentration did compound **2** even moderately increase the leakage of intracellular proteins in the PXO 86A strain (Figure 5F). Additionally, compound **2** had a slight influence on the leakage of intracellular proteins in the ES4326 strain (Figure 5D).

### 2.5. Ailanstigols A and B Lead to a Reduction in Bacterial Viability

**Figure 6:**
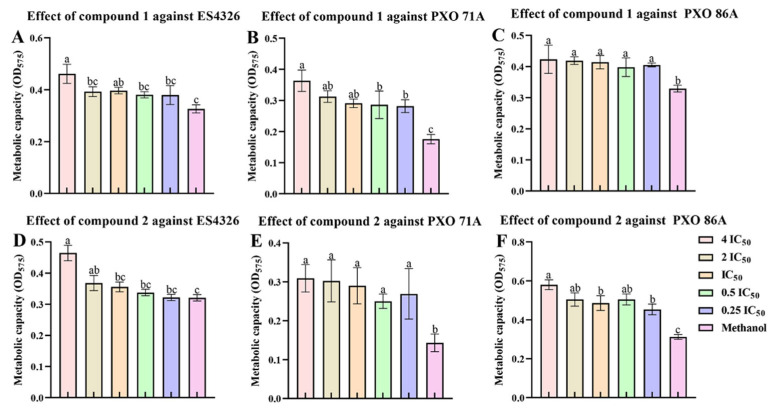
Determination of the effects of compounds **1** and **2** on the cell viability of *Xanthomonas oryzae* pv. *oryzae* PXO 71A, PXO 86A, and *Pseudomonas syringae* pv*. maculicola* ES4326. (**A**–**C**) The results of the cell viability assay on the ES4326, PXO 71A, or PXO 86A strains treated with compound **1** for 6 h. (**D**–**F**) The results of the viability assay of the ES4326, PXO 71A, or PXO 86A strain after treatment with compound **2** for 6 h. IC_50_: Half maximal inhibitory concentration. The IC_50_ values of compound **1** against the PXO 71A, PXO 86A, and ES4326 strains were determined to be 485.85 μg/mL, 314.16 μg/mL, and 289.23 μg/mL, respectively. The IC_50_ values of compound **2** against the PXO 71A, PXO 86A, and ES4326 strains were determined to be 354.09 μg/mL, 274.12 μg/mL, and 476.18 μg/mL, respectively. One-way ANOVA and Tukey’s tests were used to compare mean differences, and different lowercase letters (a, b, and c) represent significant differences (*p <* 0.05).

To investigate whether compounds **1** and **2** could reduce the viability of bacteria, *P. syringae* pv. *maculicola* and *Xanthomonas oryzae* pv. *oryzae* were cultured in the presence of **1** or **2**, and resazurin was then added to the culture mix. The resazurin was reduced by metabolic activities in live bacterial cells to the pink fluorescent substance resorufin, which allows for the assessment of bacterial metabolic activity and viability via changes in color and fluorescence. Our results showed that compound **1** reduced the bacterial cell viability of ES4326, PXO 71A, and PXO 86A strains. After the ES4326 strain was cultivated in the presence of compound **1** at a concentration of 4 times IC_50_, cell viability was reduced by 29.28 ± 3.38% compared with the solvent control group (Figure 6A). At a concentration of 4 times IC_50_, compound **1** exhibited the most apparent inhibitory effect on the cell viability of the PXO 71A strain, with an inhibition rate of 51.65 ± 4.14%. Furthermore, as the concentration increased, the bacterial cell viability decreased progressively (Figure 6B). However, compound **1** had only a slight inhibitory effect on the cell viability of the PXO 71A strain (Figure 6C). Compound **2** exhibited a certain inhibitory effect on the cell viability of the ES4326, PXO 71A, and PXO 86A strains. After the ES4326, PXO 71A, or PXO 86A strain was cultured for 6 h in the presence of compound **2** at a concentration of 4 times IC_50_, the cell viability inhibition rates were 33.85 ± 2.92%, 55.77 ± 6.97%, and 46.26 ± 2.14%, respectively (Figure 6D–F).

### 2.6. Ailanstigols A and B Inhibit the Formation of Bacterial Biofilm

**Figure 7:**
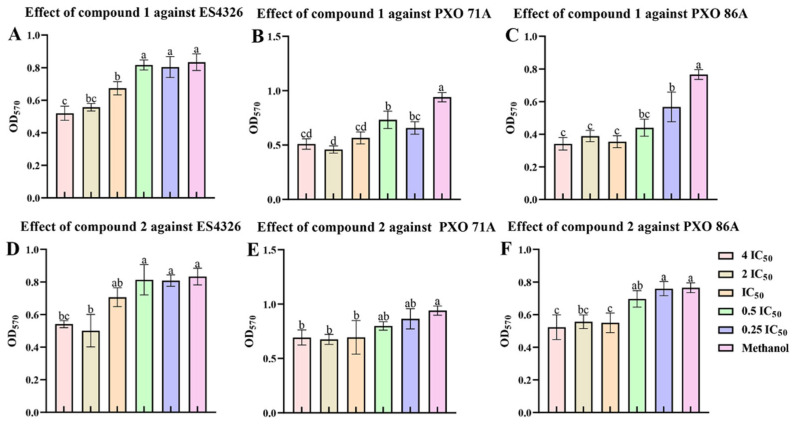
Determination of biofilm formation of *Xanthomonas oryzae* pv. *oryzae* PXO 71A, PXO 86A, and *Pseudomonas syringae* pv*. maculicola* ES4326 after treatment with compounds **1** and **2**. (**A**–**C**) The results of the biofilm formation assay in ES4326, PXO 71A, or PXO 86A treated with compound **1** for 48 h. (**D**–**F**) The results of the biofilm formation assay of ES4326, PXO 71A, or PXO 86A treated with compound **2** for 48 h. IC_50_: Half maximal inhibitory concentration. The IC_50_ values of compound **1** against the PXO 71A, PXO 86A, and ES4326 strains were determined to be 485.85 μg/mL, 314.16 μg/mL, and 289.23 μg/mL, respectively. The IC_50_ values of compound **2** against the PXO 71A, PXO 86A, and ES4326 strains were determined to be 354.09 μg/mL, 274.12 μg/mL, and 476.18 μg/mL, respectively. One-way ANOVA and Tukey’s tests were used to compare mean differences, and different lowercase letters (a, b, and c) represent significant differences (*p <* 0.05).

Bacterial biofilms are primarily composed of bacterial cells and extracellular polymeric substances (EPS), and the formation of bacterial biofilms significantly enhanced bacterial viability and resistance to antibiotics. To investigate whether compounds **1** and **2** could affect bacterial biofilm formation, these compounds were added to cultures of the ES4326, PXO 71A, or PXO 86A strain. The cultures were then stained with crystal violet to visualize the biofilm. The results demonstrated that compounds **1** and **2** at a concentration of 4 times IC_50_ could inhibit the formation of ES4326 biofilms, with inhibition rates of 37.68 ± 5.23% and 39.88 ± 11.96%, respectively, and the higher the compound concentration, the more pronounced the inhibitory effects (Figure 7A,D). At a concentration of 4 times IC_50_, compound **1** significantly inhibited the formation of PXO 71A biofilm, with an inhibition rate of 51.22 ± 3.47% (Figure 7B). However, compound **2** had only a slight influence on the formation of the PXO 71A biofilm (Figure 7B). Both compounds **1** and **2** could inhibit the formation of PXO 86A biofilms. Following treatment with compound **1** at a concentration of IC_50_, PXO 86A biofilms demonstrated the strongest inhibition, with a rate of 53.72 ± 4.72% (Figure 7C).

Specialized metabolites play a crucial role in plant defenses against pathogen infection and can inhibit the growth and pathogenicity of pathogens through various mechanisms. Steroidal hydrazones interact with the cell membranes and cell walls of *Bacillus subtilis*, *Escherichia coli*, and *Staphylococcus aureus*, disrupting bacterial structure and function. Additionally, they can inhibit bacterial metabolic pathways, suppressing bacterial growth [41]. Cationic steroids interact with bacterial cell membranes, damaging the integrity of the membrane and causing leakage of cellular contents and killing the bacteria [42]. Juglone at a concentration of 20 μg/mL is known to significantly damage the permeability and integrity of the *P. syringae* cell membrane. In addition, juglone effectively inhibits the production of extracellular polymeric substances, affecting the formation of the cell membrane and inhibiting bacterial growth [43]. In this study, steroid compounds were found that inhibited the growth of *X. oryzae* pv. *oryzae* and *P. syringae* pv. *maculicola* and reduced the cell viability of these two bacteria. After being treated with these compounds, the bacterial cell membranes underwent significant changes in both fluidity and integrity, which in turn disrupted their normal functioning and resulted in the leakage of intracellular proteins. Furthermore, as the concentration of the compounds increased, the amount of protein leakage also gradually grew, demonstrating a dose–dependent relationship. Biofilms also serve as a crucial barrier for bacteria to resist the host immune system and antibiotic activity, meaning that compounds that inhibit biofilm formation may also reduce bacterial pathogenicity. This indicates that compounds **1** and **2** inhibit the growth of invasive bacteria *X. oryzae* pv. *oryzae* and *P. syringae* pv*. maculicola* through disrupting bacterial cell membranes and inhibiting biofilm formation, providing a basis for the study of the mechanism of secondary metabolites in the prevention and control of invasive bacteria. And further investigation will be required to determine whether this antibacterial mechanism is universally applicable or to identify supplementary substances that could enhance the compound’s antibacterial activity.

## 3. Materials and Methods

### 3.1. Collection of Ailanthus altissima Leaves

The leaves of *A. altissima* were collected from the university gardens in October 2021 in Shenyang, Liaoning Province, and the samples were stored in paper bags at the College of Bioscience and Biotechnology, Shenyang Agricultural University.

### 3.2. Extraction and Isolation of Specialized Metabolites from A. altissima Leaves

First, 15.0 kg of shade-dried *A. altissima* leaves were alternately crushed and soaked in methanol three times, with each soaking process lasting at least 24 h. The resulting extraction was concentrated under a rotary evaporator to obtain the methanol extract. Then, the methanol extract was subjected multiple times to ethyl acetate solution extraction until there was no significant color change in the ethyl acetate portion. Eventually, 150 g of ethyl acetate extract was obtained. Through repeated silica gel column chromatography, sephadex LH-20 column chromatography, and HPLC semi-preparative methods, new compounds (compounds **1**–**3**) and known compounds (compounds **4**–**21**) were obtained.

Ailanstigol A (**1**):

White solid; ${\left[\alpha \right]}_{D}^{20}$ + 24.1 (*c* 0.2, MeOH); IR (KBr) *ν*_max_ 3420, 2930, 2857, 1463, 1183 cm^−1^; UV (MeOH) λ_max_ (log *ε*) 268 (2.78), 219 (3.42), 204 (3.62) nm; HR-ESI-MS *m*/*z*: 483.3816 [M + Na]^+^_._ ^1^H and ^13^C NMR data, see Table 1.

Ailanstigol B (**2**):

White solid, IR (KBr) *ν*_max_ 3439, 2837, 1519, 1410, 1135, 1061, 827 cm^−1^; UV (MeOH) λ_max_ (log *ε*) 210 (3.02), 198 (2.41) nm; HR-ESI-MS *m*/*z*: 469.3644 [M + Na]^+^_._ ^1^H and ^13^C NMR data, see Table 1.

(2′*R*,3′*R*)-7-(2′,3′,6′-trihydroxy-3′-methylhexyloxy)-6,8-dimethoxycoumarin (**3**):

White powder; ${\left[\alpha \right]}_{D}^{22}$ + 35.2 (*c* 0.1, MeOH); IR (KBr) *ν*_max_ 3327, 2963, 1635, 1527, 1435, 1328, 1219, 1031, 898 cm^−1^; UV (MeOH) λ_max_ (log *ε*) 337 (3.71), 295 (3.88), 222 (4.22), 207 (4.49) nm; HR-ESI-MS *m*/*z*: 391.1371 [M + Na]^+^_._ ^1^H and ^13^C NMR data, see Table 2.

### 3.3. Preparation of NA and KB Culture Media

NA Medium: 3 g of beef extract, 1 g of yeast extract, 5 g of peptone, and 10 g of sucrose were mixed together, then diluted to 1000 mL with water. If a solid medium was required, 20 g of agar powder was added.

KB Medium: 1.5 g of potassium dihydrogen phosphate, 1.5 g of magnesium sulfate, 20 g of peptone, and 10 mL of glycerol were mixed together, then diluted to 1000 mL. If a solid medium was required, 15 g of agar powder was added to the mixture.

### 3.4. ECD Spectra Calculations

A J-1500 circular dichroism spectrophotometer (JASCO, Japan) was used to detect the CD curves of compounds **1**, **2**, and **3**, and these calculated conformational isomers were optimized at the B3LYP/6-311G(d, *p*) level using Gaussian 16 W and GaussView 6.0. The ECD spectra were subsequently generated via SpecDis 1.7 software [44].

### 3.5. HPLC Analysis

To verify that compound **1** is a natural product rather than a transformation of compound **2**, we separately immersed 50 g of *A. altissima* leaves in 75% ethanol, methanol, acetone, and dichloromethane solvents. After 24 h, the extracts obtained from each solvent were concentrated using a rotary evaporator (IKA, Staufen, Germany). Subsequently, the chemical components of the extracts were qualitatively and quantitatively analyzed by HPLC-DAD (Agilent, Santa Clara, CA, USA). The sample was dissolved in methanol and separated at a flow rate of 1 mL/min on an Eclipse XDB-C18 (5 μm, 4.6 × 250 mm) chromatographic column. The mobile phase consisted of HPLC-grade water (A) and methanol (B), with the following gradient elution program: from 0 to 30 min, B increased linearly from 5% to 95%; from 30 to 40 min, B was maintained at 95%. The column oven temperature was set to 35 °C, and detection was performed over the full wavelength range of 200–400 nm. In addition, compound **2** was dissolved in methanol to prepare a solution with a final concentration of 1.5 mg/mL. Quantitative analysis of the content of compound **2** in the samples was conducted after standing for 24 h and 48 h, respectively.

### 3.6. Activation of Bacteria

The strains used in the experiment were *X. oryzae* pv. *oryzae* (PXO 71A and PXO 86A) and *P. syringae* pv. *maculicola* ES4326 provided by Fudan University. Using plate streak separation methods [45], the PXO 71A and PXO 86A strains preserved in glycerol were cultured on NA solid medium, while *P. syringae* pv. *maculicola* ES4326 was cultured on KB solid medium. When the single cultured colonies had been picked, they were inoculated into the corresponding NA and KB liquid media and incubated overnight in an incubator for 24 h–48 h (28 °C, 180 rpm) to prepare a bacterial stock solution with an OD_600_ value of 0.3–0.4.

### 3.7. Testing for Antimicrobial Activity in the Isolated Compounds

The activity of the compounds against different strains of bacteria was tested using a double dilution method [46]. Bacterial suspensions with an OD_600_ value of 0.3–0.4 in NA or KB liquid media were diluted approximately 10 times. The test compound was dissolved in methanol and prepared as a stock solution with a concentration of 2048 μg/mL. In a 96-well plate, 1 μL of the compound solution and 199 μL of the diluted bacterial suspension were added to each well, resulting in a final concentration range of the compound in each well from 512 to 16 μg/mL. Three biological replicates were set up for each concentration. The positive controls were kanamycin or streptomycin at concentrations of 128–4 μg/mL. The negative controls consisted of 1 μL of methanol, 99 μL of NA or KB liquid medium, and 100 μL of bacterial suspension. The blank control was 200 μL of NA or KB liquid medium. The 96-well plate was then placed in a 28 °C incubator, and sample absorbance at 600 nm was measured every 12 h using an enzyme-linked immunosorbent assay (ELISA) reader.

### 3.8. Bacterial Protein Leakage Assay

Coomassie brilliant blue was used to determine the amount of leakage of bacterial intracellular proteins [47]. Briefly, in sterilized 1.5 mL centrifuge tubes, the tested compound was prepared in 100 μL drug-containing media at concentrations of 4 IC_50_, 2 IC_50_, IC_50_, 0.5 IC_50_, or 0.25 IC_50_. A solvent control was also set up. Next, 100 μL of bacterial suspension was added to each tube, and the samples were then incubated in an orbital shaker at 28 °C for 6 h and then centrifuged at 5000 rpm for 3 min. Then, 20 μL of supernatant was taken, and Coomassie brilliant blue was added to stain the proteins. The sample OD_595_ value was then measured using a Multiskan FC (Thermo, Shanghai, China). The standard curve y = 0.0125x + 3.823 was used to determine the content of protein.

### 3.9. Bacterial Viability Assay

Bacterial viability was assayed using resazurin [48]. First, 100 μL of culture medium, 99 μL of bacterial suspension, and 1 μL of test compound were added to each well of a 96-well plate to final compound concentrations of 4 IC_50_, 2 IC_50_, IC_50_, 0.5 IC_50,_ and 0.25 IC_50_, and plates were then incubated at 28 °C for 6 h. Then, 20 μL of 5% resazurin was added to each sample, and samples were again incubated at 28 °C for 1 h. An equal amount of methanol was used as a negative control and added to the same bacterial suspension. An ELISA reader was then used to measure sample absorbance at 575 nm.

### 3.10. Bacterial Biofilm Inhibition Assay

Bacterial biofilm inhibition was assayed using crystal violet staining [49]. Briefly, the test compound was prepared into drug-containing media to a final concentration of 4 IC_50_, 2 IC_50_, IC_50_, 0.5 IC_50_, or 0.25 IC_50_ in a volume of 3 mL and thoroughly mixed. Next, in a 96-well plate, 179 μL of drug-containing culture medium, 1 μL of compound, and 20 μL of bacterial suspension were added, and samples were incubated at 28 °C for 48 h. An equal amount of methanol was used as a negative control. Samples were then washed twice with diluted water, after which crystal violet (1%) was added, and the samples were left to stand for 30 min. The floating color was washed away with diluted water, then anhydrous ethanol was added, and the samples were left to stand for a further 30 min. An ELISA reader was then used to measure sample absorbance at 570 nm.

### 3.11. Data Analysis

The measured data were processed in GraphPad Prism 5.0 to obtain line graphs. In order to determine the relationship between test compound concentration and bacterial growth inhibition rate, statistical analysis of the data was performed using SPSS 20.0, and the Probit analysis method in SPSS was used to calculate the IC_50_. The data were presented as the SD of biological replicates. The treatment groups at different times and concentrations were compared with their corresponding control groups, then an independent samples *t*-test was used to compare the experimental groups with the control groups, where *p* < 0.05 is “*”, *p* < 0.01 is “**”, and *p* < 0.001 is “***”. Univariate analysis of ANOVA (Tukey’s Honestly Significant Difference, *p* < 0.05) was used to compare quantitative data among three or more groups.

## 4. Conclusions

This study systematically isolated, purified, and identified 21 compounds from the leaves of *A. altissima*, including two new steroids and one new coumarin, which demonstrate the ample chemical diversity in *A. altissima*. Further screening of the antimicrobial activity of the two new steroids revealed that ailanstigols A (**1**) and B (**2**) exhibited varying degrees of inhibitory activity against the invasive *Xanthomonas oryzae* PXO 71A and PXO 86A, as well as *Pseudomonas syringae* pv. *maculicola*. Both compounds **1** and **2** showed the highest inhibitory activity against PXO 86A. After the bacteria were cultured in the presence of compounds **1** and **2**, the compounds induced protein leakage and a decline in bacterial cell viability, effectively inhibited biofilm formation, and suppressed the growth of the bacteria. Therefore, we propose the utilization of the two new steroids from *A. altissima* leaves as potential natural candidate pesticides, providing better opportunities for further innovation and development of environmentally friendly antimicrobial compounds.

## Figures and Tables

**Table 1:** The ^1^H (600 MHz) and ^13^C (150 MHz) NMR data of compounds **1** and **2** in methanol-*d*_4_ (*δ*, ppm).

Position	Compound 1		Compound 2	
	*δ*_H_ (*J*[Hz])	*δ*_C_	*δ*_H_ (*J*[Hz])	*δ*_C_
1	1.10, m 1.84, m	38.1, t	1.04, m 1.86, m	38.2, t
2	1.48, m 1.88, m	32.1, t	1.48, m 1.78, m	32.3, t
3	3.45, m	72.2, d	3.41, m	72.1, d
4	2.29, m	43.1, t	2.24, m	42.5, t
5	-	148.0, s	-	144.1, s
6	5.77, d (4.1)	121.5, d	5.27, br s	127.4, d
7	3.34, br s	75.2, d	3.72, br d (8.2)	73.7, d
8	1.50, m	38.0, d	1.45, m	40.7, d
9	1.28, m	44.1, d	1.02, m	50.0, d
10	-	38.6, s	-	37.6, s
11	1.53, m	21.9, t	1.54, m	22.2, t
12	1.17, m 2.05, m	41.1, t	1.20, m 2.09, m	41.5, t
13	-	43.5, s	-	44.3, s
14	1.49, m	50.6, d	1.13, m	58.0, d
15	1.15, m 1.33, m	24.8, t	1.24, m 1.44, m	27.0, t
16	1.16, m	25.5, t	1.13, m	25.4, t
17	1.48, m	59.1, d	1.44, m	58.7, d
18	0.85, s	13.7, q	0.87, s	14.1, q
19	0.99, s	18.8, q	1.06, s	19.4, q
20	-	76.2, s	-	76.2, s
21	1.24, s	26.2, q	1.24, s	26.2, q
22	1.52, m	43.4, t	1.28, m 1.51, m	43.4, t
23	1.67, m	23.4, t	1.65, m 1.79, m	23.8, t
24	0.94, m	47.7, d	0.93, m	47.7, d
25	1.69, m	30.4, d	1.69, m	30.4, d
26	0.87, d (6.5)	20.1, q	0.87, d (6.6)	20.0, q
27	0.86, d (6.5)	19.6, q	0.86, d (6.6)	19.6, q
28	1.66, m	24.1, t	1.33, m	24.1, t
29	0.89, t (7.3)	12.4, q	0.88, t (7.3)	12.4, q
7-OMe	3.35, s	56.9, q		

**Table 2:** The ^1^H (600 MHz) and ^13^C (150 MHz) NMR data of compound **3** in acetone-*d*_6_ (*δ*, ppm).

Position	Compound 3	
	*δ*_H_ (*J*[Hz])	*δ*_C_
2	-	160.4, s
3	6.34, d (9.5)	115.7, d
4	7.92, d (9.5)	144.7, d
5	7.10, s	105.6, d
6	-	150.9, s
7	-	145.9, s
8	-	141.8, s
9	-	143.7, s
10	-	115.6, s
1′	4.08, dd (10.3, 8.3); 4.54, dd (10.3, 2.6)	77.0, t
2′	3.80, dd (8.3, 2.6)	76.1, d
3′	-	73.3, s
4′	1.55, m; 1.68, m	36.6, t
5′	1.65, m	27.3, t
6′	3.53, m	63.2, t
7′	1.13, s	22.8, q
6-OMe	3.90, s	56.7, q
8-OMe	4.00, s	62.0, q

## Data Availability

The data are contained within the article and Supplementary Materials.

## Supplementary Materials

The following supporting information can be downloaded at https://www.mdpi.com/article/10.3390/molecules30122576/s1: Chemical structures of known compounds (Figure S1); growth inhibitory activity of compounds **13**, **15**, and **17** (Figure S2); qualitative graphs of the effects of compounds **1** and **2** on cell viability and biofilm formation (Figures S3 and S4); 1D and 2D NMR spectra, HR-ESI-MS, optical rotation data of compounds **1**–**3** (Figures S5–S28); qualitative and quantitative analysis of compounds **1** and **2** (Figures S29 and S30); and growth inhibitory activity of kanamycin and streptomycin (Figure S31).
