# Supplementary material for: New Steroids Obtained from Ailanthus altissima Leaves Inhibit the Invasive Bacteria Xanthomonas oryzae pv. oryzae and Pseudomonas syringae pv. maculicola
Source: Molecules. 2025 Jun 13;30(12):2576. doi: 10.3390/molecules30122576 (PMC12195840; doi:10.3390/molecules30122576)
Supplement: Supplementary file 1 [file molecules-30-02576-s001.zip › molecules-3642434-supplementary.pdf]

New steroids obtained from *Ailanthus altissima* leaves inhibit the invasive bacteria *Xanthomonas oryzae* pv. *oryzae* and *Pseudomonas syringae* pv. *maculicola*

Supporting information

Yuhong Yang †, Yue Wu †, Zhengyi Gao, Zhixiang Liu, Juan Hua and Shihong Luo *

Engineering Research Center of Protection and Utilization of Plant Resources, College of Bioscience and Biotechnology, Shenyang Agricultural University, Shenyang 110866, China

*Corresponding Authors: Prof. Shihong Luo, E-mail address: luoshihong@syau.edu.cn (http://orcid.org/0000-0003-3500-3466)

†These authors contributed equally to this work.

Table of Contents

Figure S1. The chemical structures of known compounds **4–21** isolated from *A. altissima* leaves **Error! Bookmark not defined.**

Figure S2. Growth inhibitory activity of compounds **13**, **15**, and **17** against invasive agricultural bacteria S5

Figure S3. The qualitative effects of compounds **1** and **2** on the cell viability of *P. syringae* pv. *maculicola*, PXO 71A, and PXO 86A **Error! Bookmark not defined.**

Figure S4. The qualitative effects of compounds **1** and **2** on biofilm formation in the bacteria *P. syringae* pv. *maculicola*, PXO 71A, and PXO 86A........ S7

Figure S5. ^{1}H NMR spectrum of compound **1** in methanol-d_4 .... S8

Figure S6. ^{13}C NMR and DEPT spectra of compound **1** in methanol-d_4........ S8

Figure S7. HSQC spectrum of compound **1** in methanol-d_4........ S9

Figure S8. HMBC spectrum of compound **1** in methanol-d_4........ S9

Figure S9. ^{1}H-^{1}H COSY spectrum of compound **1** in methanol-d_4 .... S10

Figure S10. ROESY spectrum of compound **1** in methanol-d_4........ S10

Figure S11. HR-ESI-MS spectrum of compound **1** S11

Figure S12. IR spectrum of compound **1** S12

Figure S13. Optical rotation data of compound **1** S13

Figure S14. ^{1}H NMR spectrum of compound **2** in methanol-d_4 .... S14

Figure S15. ^{13}C NMR and DEPT spectra of compound **2** in methanol-d_4........ S14

Figure S16. HSQC spectrum of compound **2** in methanol-d_4........ S15

Figure S17. HMBC spectrum of compound **2** in methanol-d_4........ S15

Figure S18. ^{1}H-^{1}H COSY spectrum of compound **2** in methanol-d_4 .... S16

Figure S19. ROESY spectrum of compound **2** in methanol-d_4........ S16

Figure S20. HR-ESI-MS spectrum of compound **2** S17

Figure S21. ^{1}H NMR spectrum of compound **3** in acetone-d_6........ S18

Figure S22. ^{13}C NMR and DEPT spectra of compound **3** in acetone-d_6 .... S18

Figure S23. HSQC spectrum of compound **3** in acetone-d_6 .... S19

Figure S24. HMBC spectrum of compound **3** in acetone-d_6 .... S19

Figure S25. ^{1}H-^{1}H COSY spectrum of compound **3** in acetone-d_6 .... S20

Figure S26. ROESY spectrum of compound **3** in acetone-d_6 .... S20

Figure S27. HR-ESI-MS spectrum of compound **3** S21

Figure S28. Optical rotation data of compound **3** S22

Figure S29. Qualitative and quantitative analysis of compounds **1** and **2** in *A. altissima* leaves S23

Figure S30. HPLC analysis chromatograms of compound **2** in methanol at 0, 24 and 48 hours S24

Figure S31. Growth inhibitory activity of kanamycin and streptomycin against invasive agricultural bacteria S25

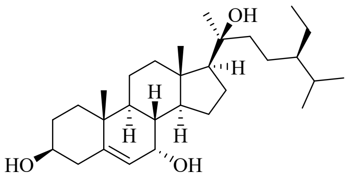

Compound **4**

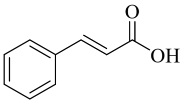

Compound **5**

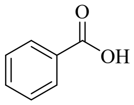

Compound **6**

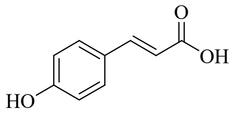

Compound **7**

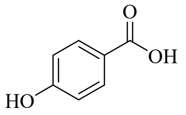

Compound **8**

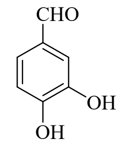

Compound **9**

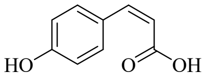

Compound **10**

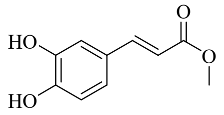

Compound **11**

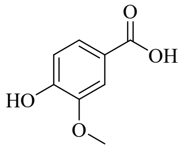

Compound **12**

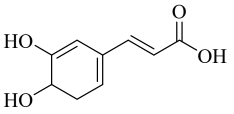

Compound **13**

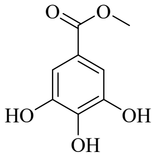

Compound **14**

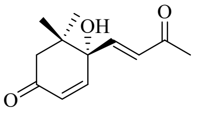

Compound **15**

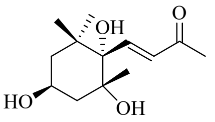

Compound **16**

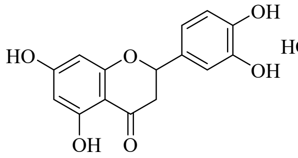

Compound **17**

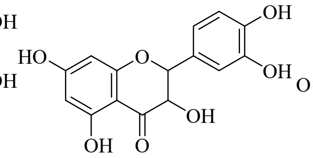

Compound **18**

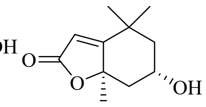

Compound **19**

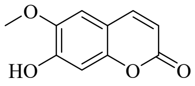

Compound **20**

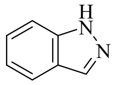

Compound **21**

Figure S1. The chemical structures of known compounds **4–21** isolated from *A. altissima* leaves.

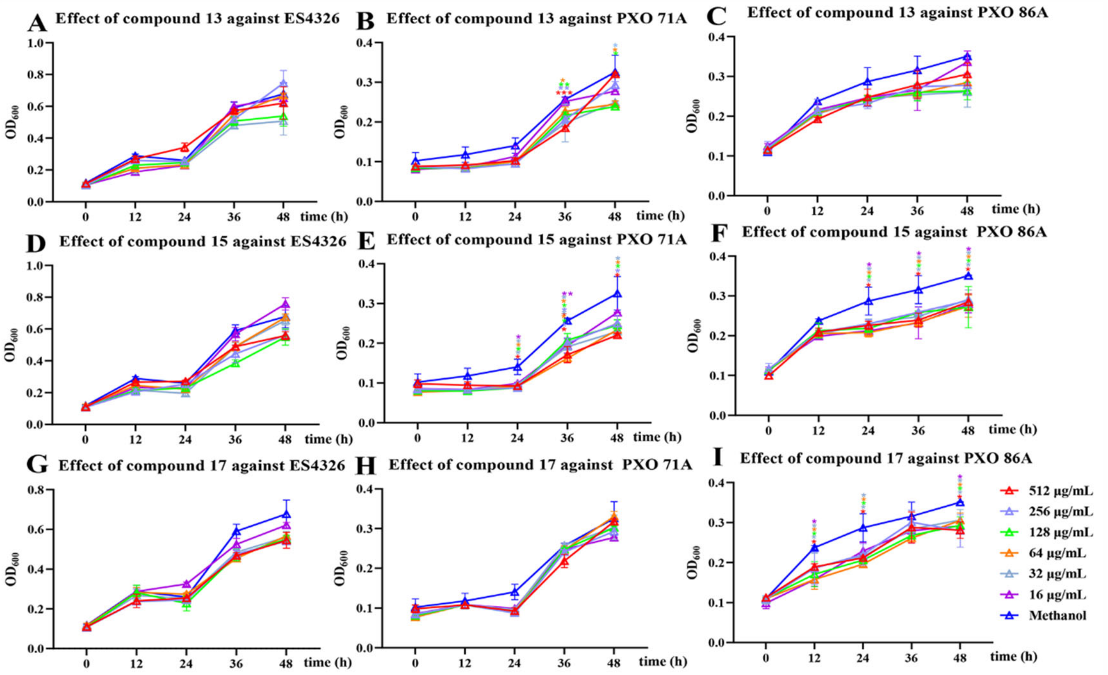

Figure S2. Growth inhibitory activity of compounds **13**, **15**, and **17** against invasive agricultural bacteria. (A–C) represent the results of the inhibitory activity screening of compound **13** against the bacteria *P. syringae* pv. *maculicola*, PXO 71A, and PXO 86A. (D–F) represent the results of the inhibitory activity screening of compound **15** against the bacteria *P. syringae* pv. *maculicola*, PXO 71A, and PXO 86A. (G–I) represent the results of the inhibitory activity screening of compound **17** against the bacteria *P. syringae* pv. *maculicola*, PXO 71A, and PXO 86A. An independent samples **t**-test was used to compare the experimental groups with the control groups, where $p < 0.05$ was "*", $p < 0.01$ was "**", and $p < 0.001$ was "***".

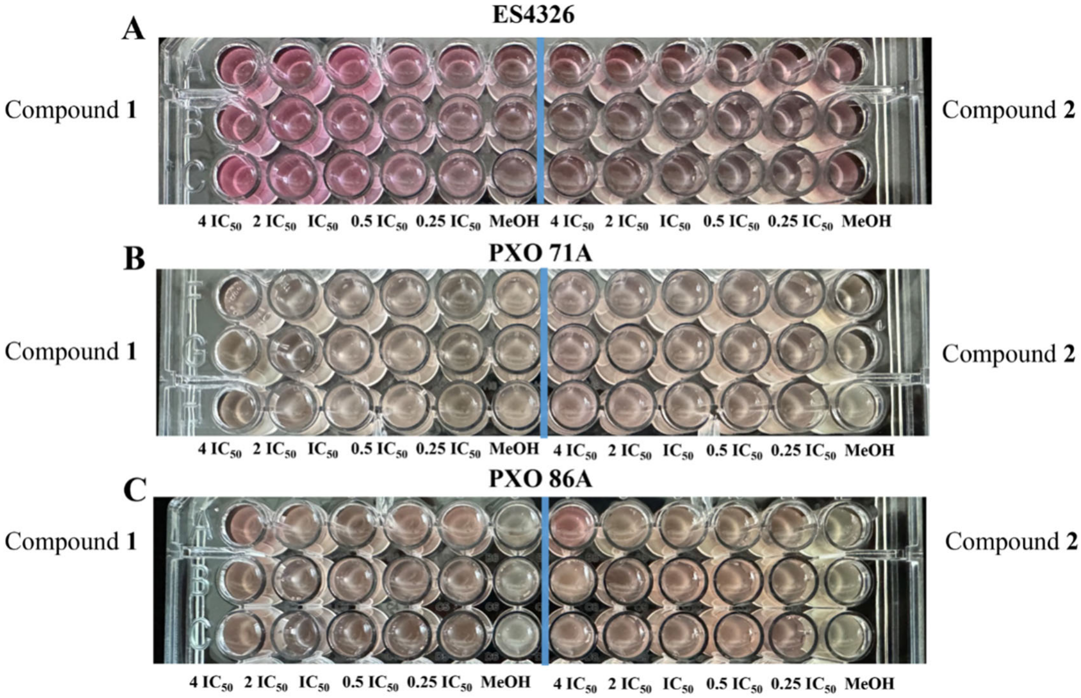

Figure S3. The qualitative effects of compounds **1** and **2** on the cell viability of *P. syringae* pv. *maculicola*, PXO 71A, and PXO 86A. Figure **A** shows the qualitative assessment of the effects of compounds **1** and **2** on the cell viability of *P. syringae* pv. *maculicola*. Figure B shows the qualitative assessment of the effects of compounds **1** and **2** on the cell viability of PXO 71A. Figure C shows the qualitative assessment of the effects of compounds **1** and **2** on the cell viability of PXO 86A.

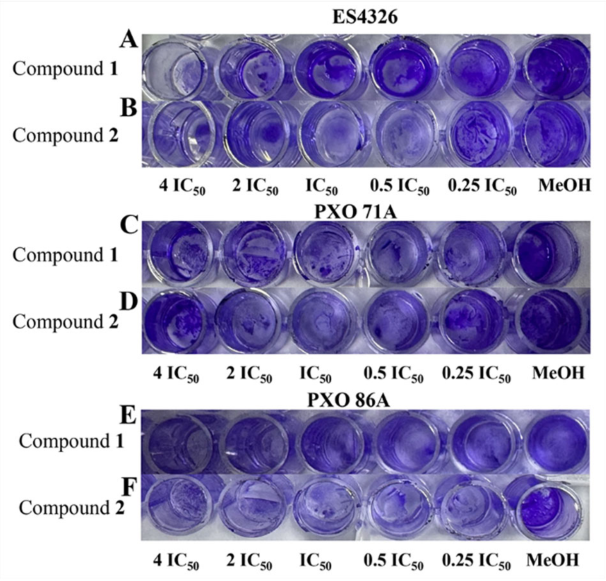

Figure S4. The qualitative effects of compounds **1** and **2** on biofilm formation in the bacteria *P. syringae* pv. *maculicola*, PXO 71A, and PXO 86A. Figure **A** presents a qualitative assessment of the effect of compound **1** on biofilm formation in *P. syringae* pv. *maculicola*. Figure **B** presents a qualitative assessment of the effect of compound **2** on biofilm formation in *P. syringae* pv. *maculicola*. Figure **C** presents a qualitative assessment of the effect of compound **1** on biofilm formation in PXO 71A. Figure **D** presents a qualitative assessment of the effect of compound **2** on biofilm formation in PXO 71A. Figure **E** presents a qualitative assessment of the effect of compound **1** on biofilm formation in PXO 86A. Figure **F** presents a qualitative assessment of the effect of compound **2** on biofilm formation in PXO 86A.

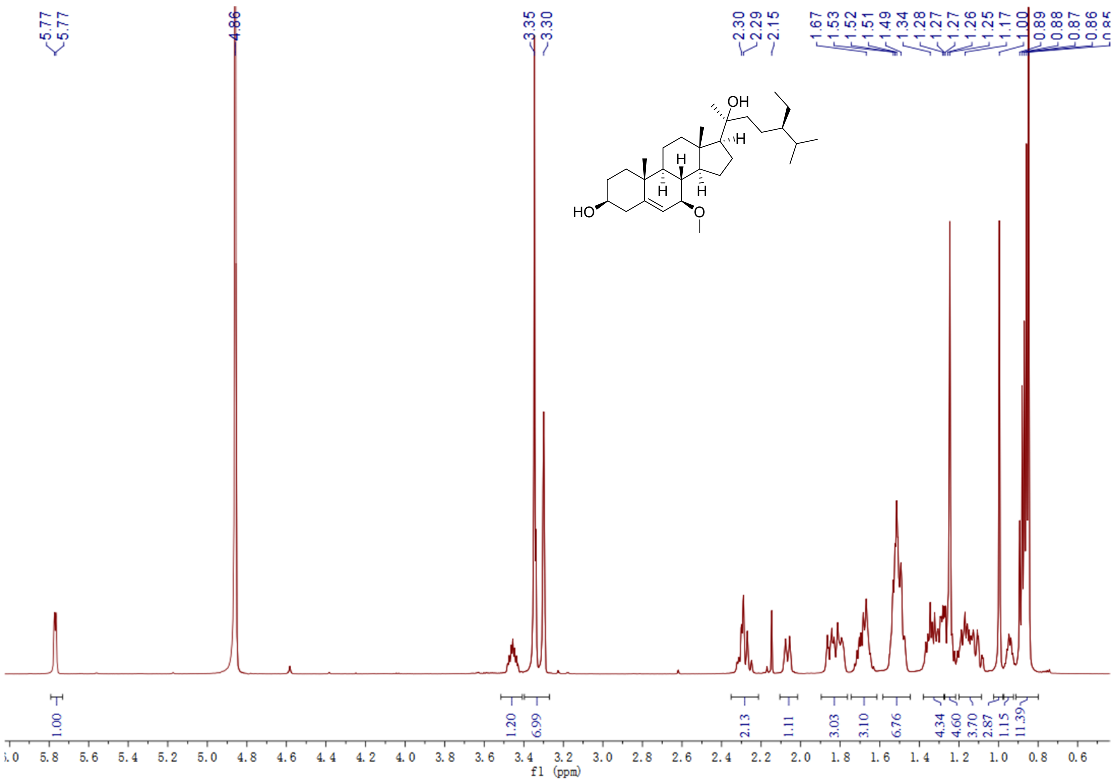

Figure S5. ^{1}H NMR spectrum of compound **1** in methanol-*d*4.

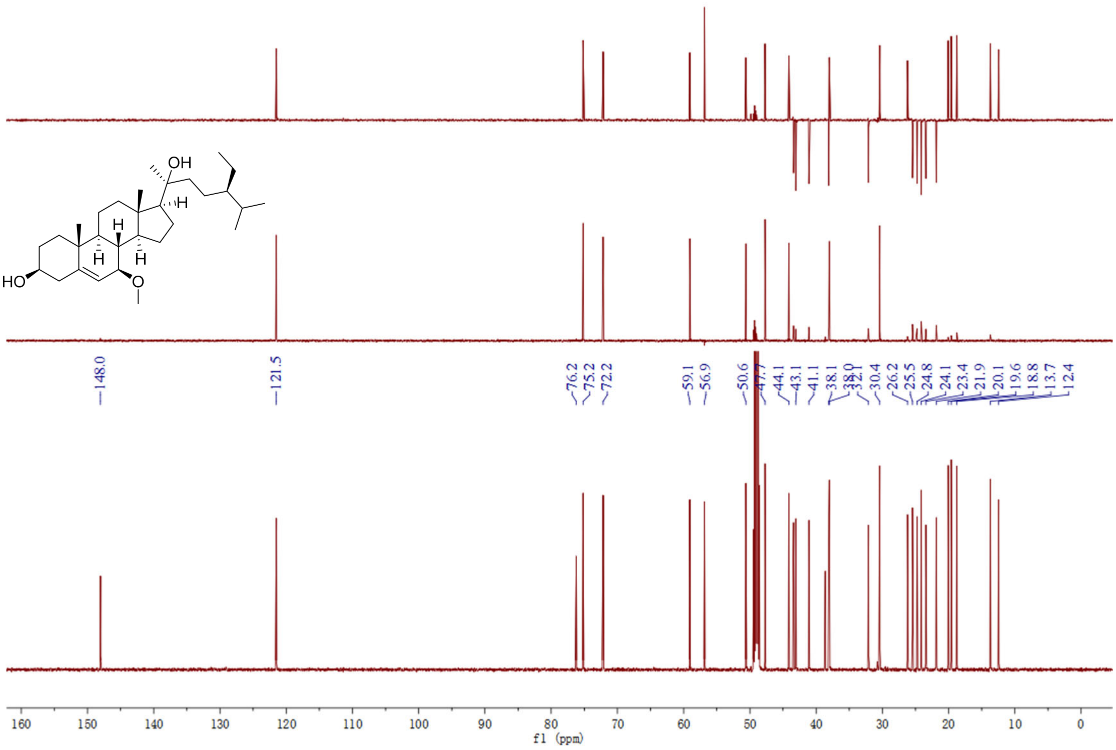

Figure S6. ^{13}C NMR and DEPT spectra of compound **1** in methanol-*d*4.

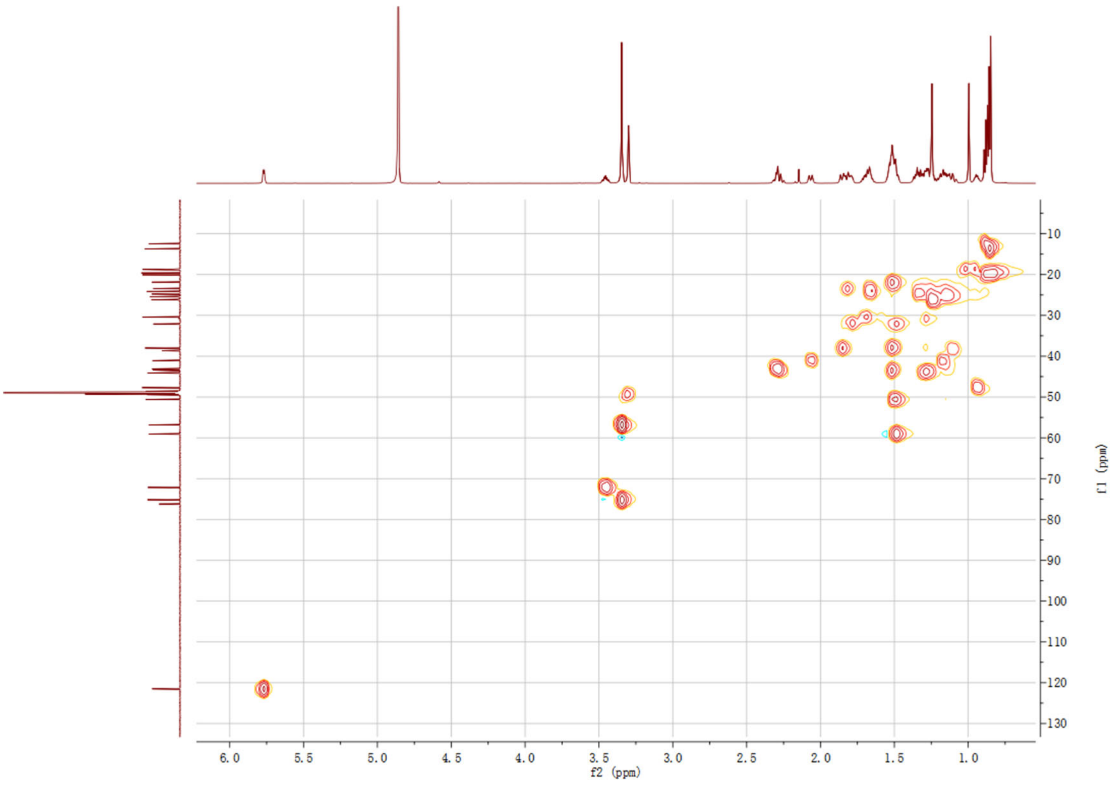

Figure S7. HSQC spectrum of compound **1** in methanol-d_4.

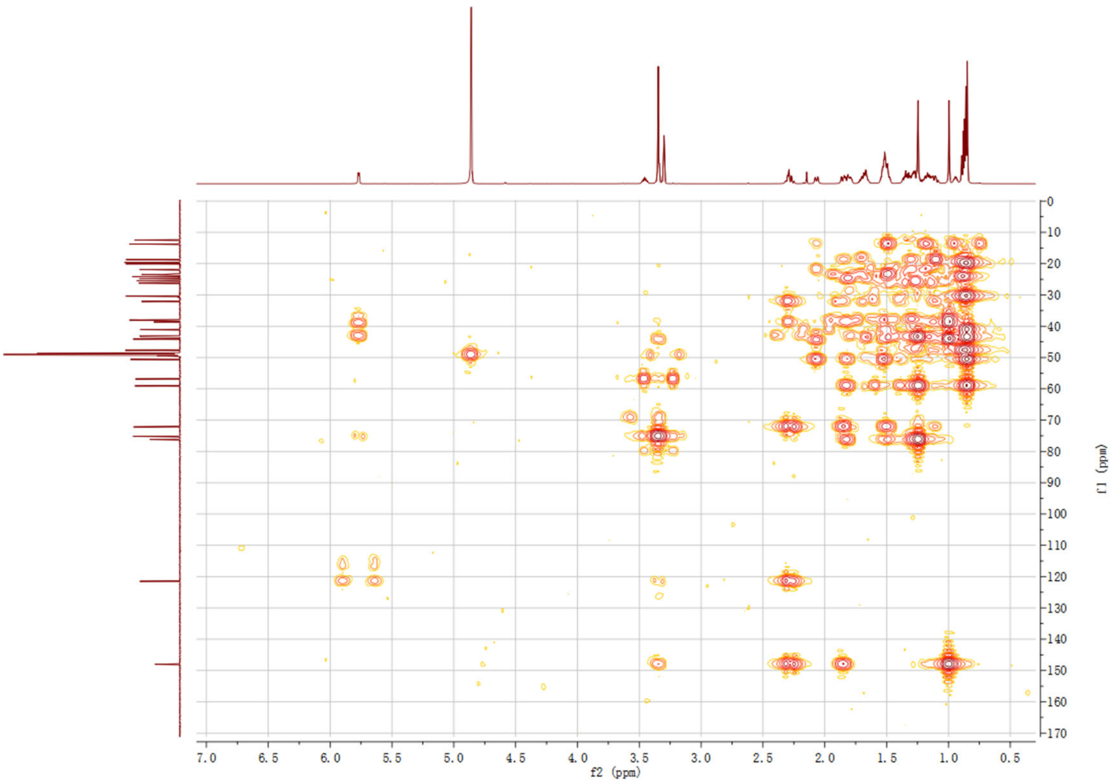

Figure S8. HMBC spectrum of compound **1** in methanol-d_4.

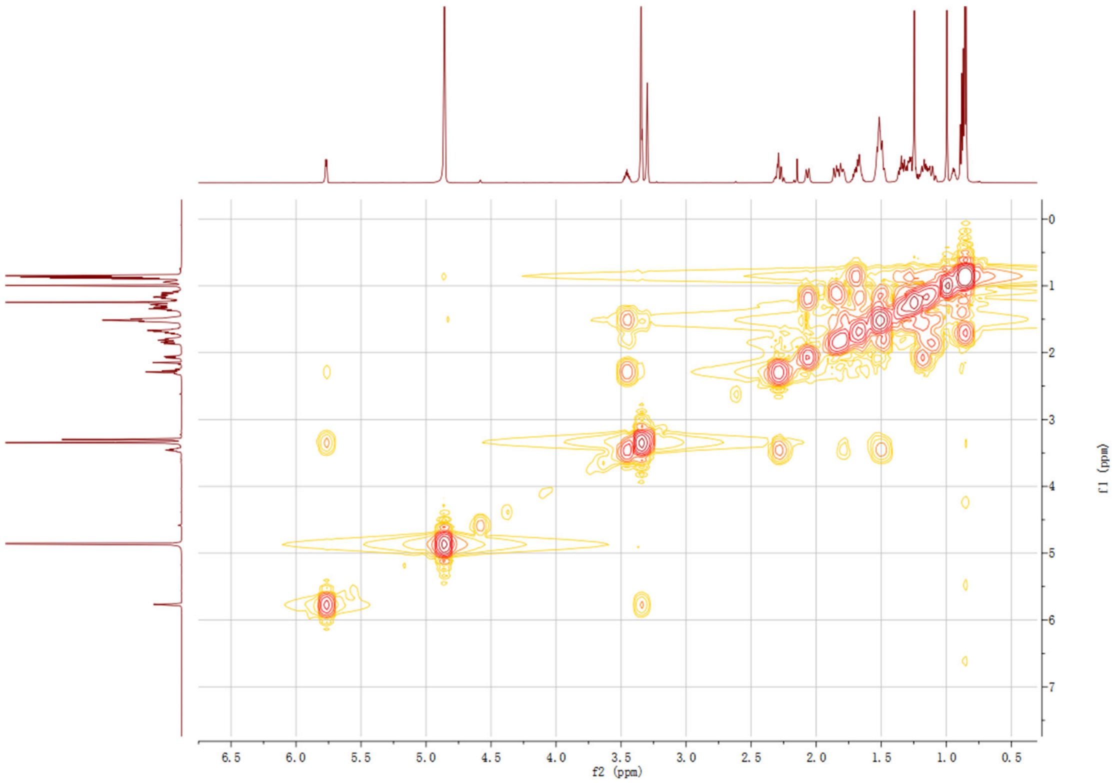

Figure S9. ^1H-^1H COSY spectrum of compound **1** in methanol-d_4.

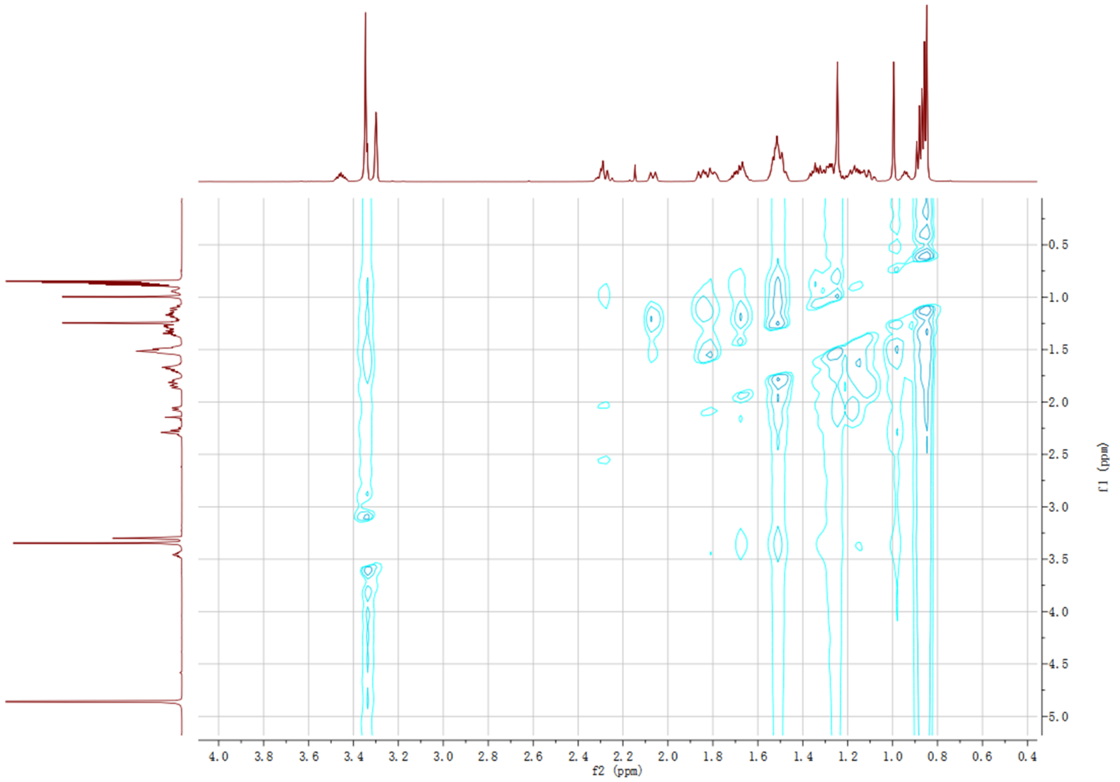

Figure S10. ROESY spectrum of compound **1** in methanol-d_4.

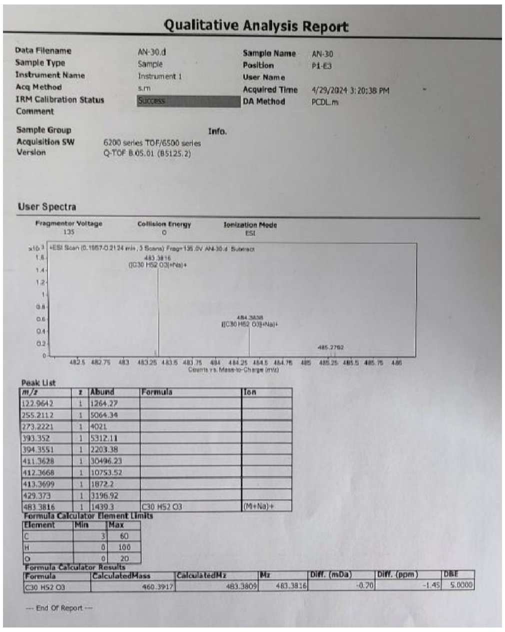

Qualitative Analysis Report

Data Filename AN-30.d
Sample Type Sample
Instrument Name Instrument 1
Acq Method s.m
IRM Calibration Status Success
Comment
Sample Name AN-30
Position P1-E3
User Name
Acquired Time 4/29/2024 3:20:38 PM
DA Method PCDL.m

Sample Group
Info.
Acquisition SW 6200 series TOF/6500 series
Version Q-TOF B.05.01 (B5125.2)

User Spectra

Fragmentor Voltage 135
Collision Energy 0
Ionization Mode ESI

Peak List

m/z	z	Abund	Formula	Ion
122.9642	1	1264.27		
255.2112	1	5064.34		
273.2221	1	4021		
393.352	1	5312.11		
394.3551	1	2203.38		
411.3628	1	30496.23		
412.3668	1	10753.52		
413.3699	1	1872.2		
429.373	1	3196.92		
483.3816	1	1439.3	C30 H52 O3	(M+Na)+

Formula Calculator Element Limits

Element	Min	Max
C	3	60
H	0	100
O	0	20

Formula Calculator Results

Formula	CalculatedMass	CalculatedMz	Mz	Diff. (mDa)	Diff. (ppm)	DBE
C30 H52 O3	460.3917	483.3809	483.3816	-0.70	-1.45	5.0000

--- End Of Report ---

Figure S11. HR-ESI-MS spectrum of compound **1**.

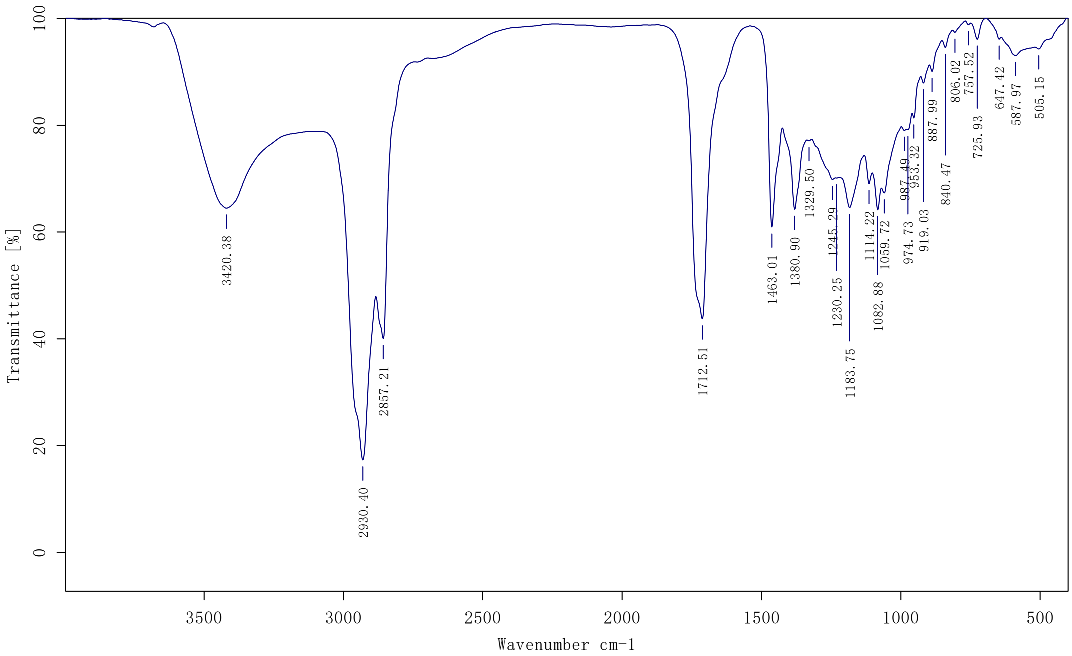

Figure S12. IR spectrum of compound **1**.

Rudolph Research Analytical

This sample was measured on an Autopol VI, Serial #91058
Manufactured by Rudolph Research Analytical, Hackettstown, NJ, USA.

Measurement Date : Tuesday, 04-JUN-2024

Set Temperature : 25.0

Time Delay : Disabled

Delay between Measurement : Disabled

n	Average	Std.Dev.	% RSD	Maximum	Minimum
5	-20.45	0.10	-0.48	-20.34	-20.52

S.No	Sample ID	Time	Result	Scale	OR °Arc	WLG.nm	Lg.mm	Conc.g/100ml	Temp.
1	AN-30	10:48:41 AM	-20.52	SR	-0.119	589	100.00	0.580	25.0
2	AN-30	10:48:47 AM	-20.52	SR	-0.119	589	100.00	0.580	25.0
3	AN-30	10:48:54 AM	-20.34	SR	-0.118	589	100.00	0.580	25.0
4	AN-30	10:49:00 AM	-20.52	SR	-0.119	589	100.00	0.580	25.0
5	AN-30	10:49:06 AM	-20.34	SR	-0.118	589	100.00	0.580	25.0

Figure S13. Optical rotation data of compound **1**.

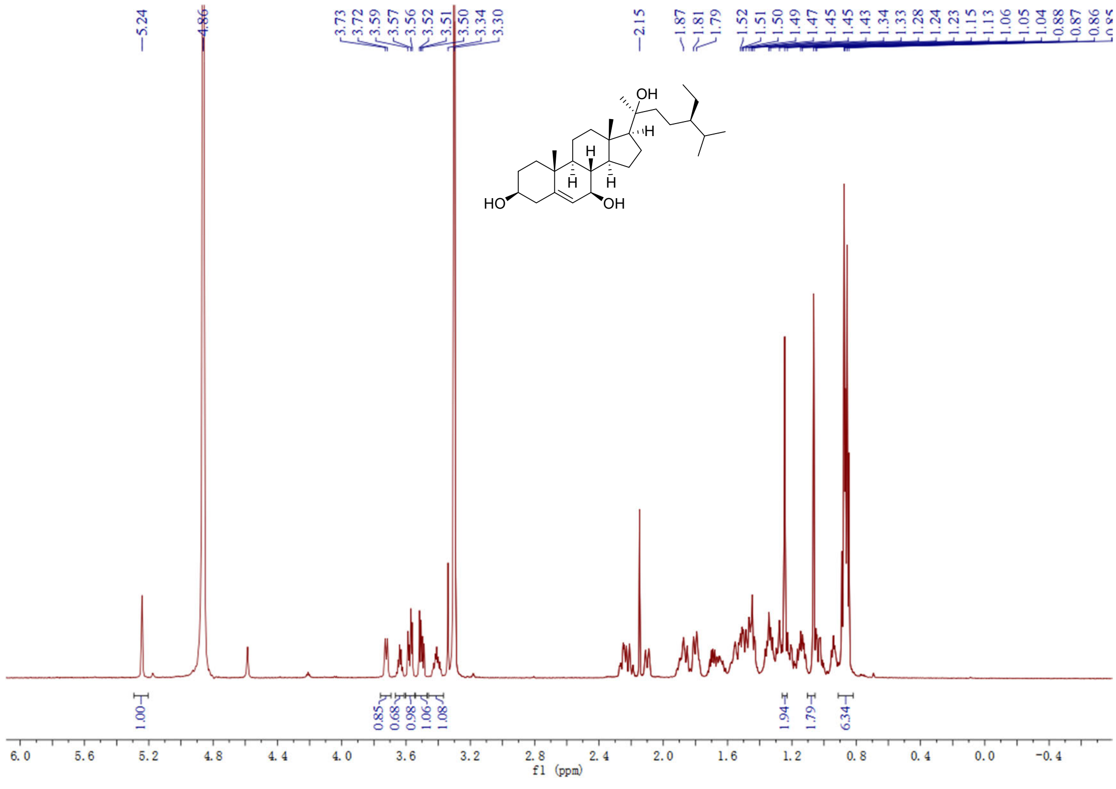

Figure S14. ^{1}H NMR spectrum of compound **2** in methanol-*d*4.

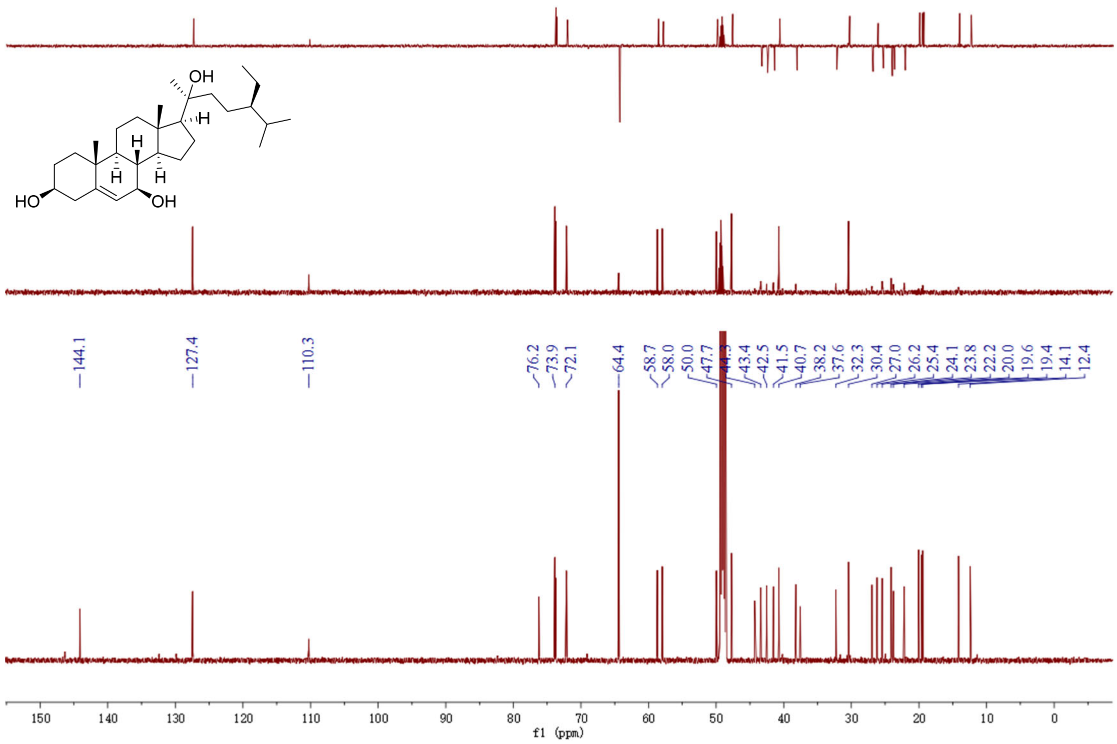

Figure S15. ^{13}C NMR and DEPT spectra of compound **2** in methanol-*d*4.

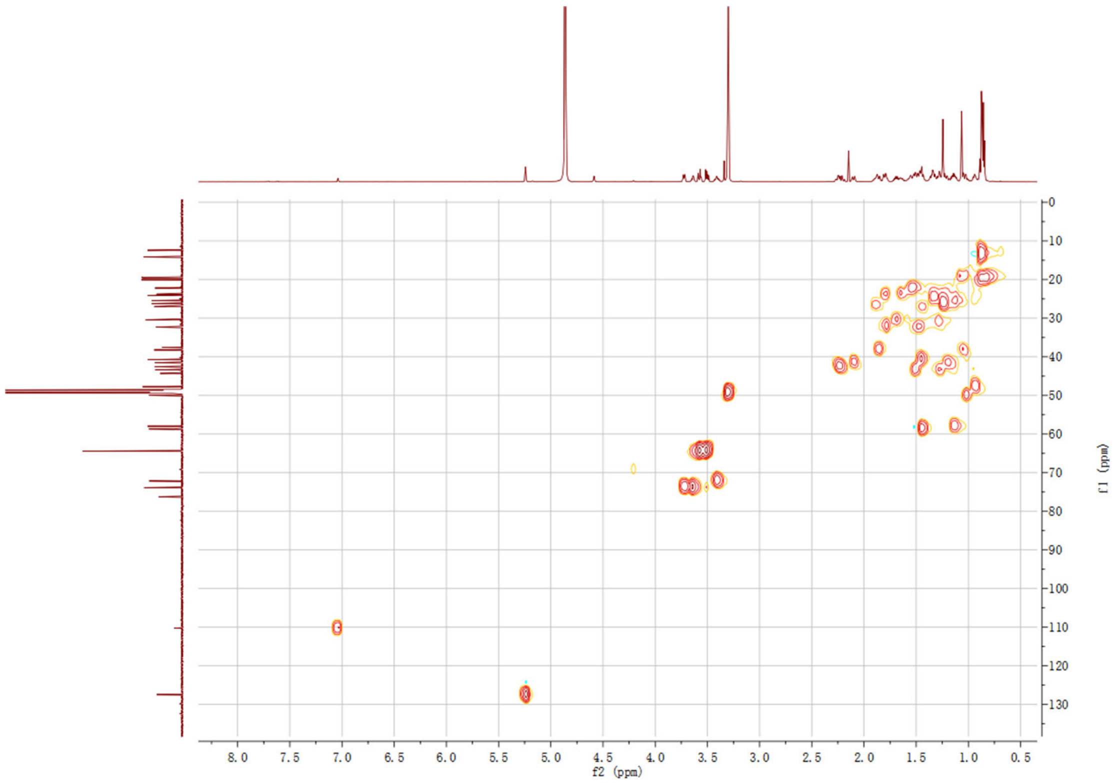

Figure S16. HSQC spectrum of compound **2** in methanol-*d*4.

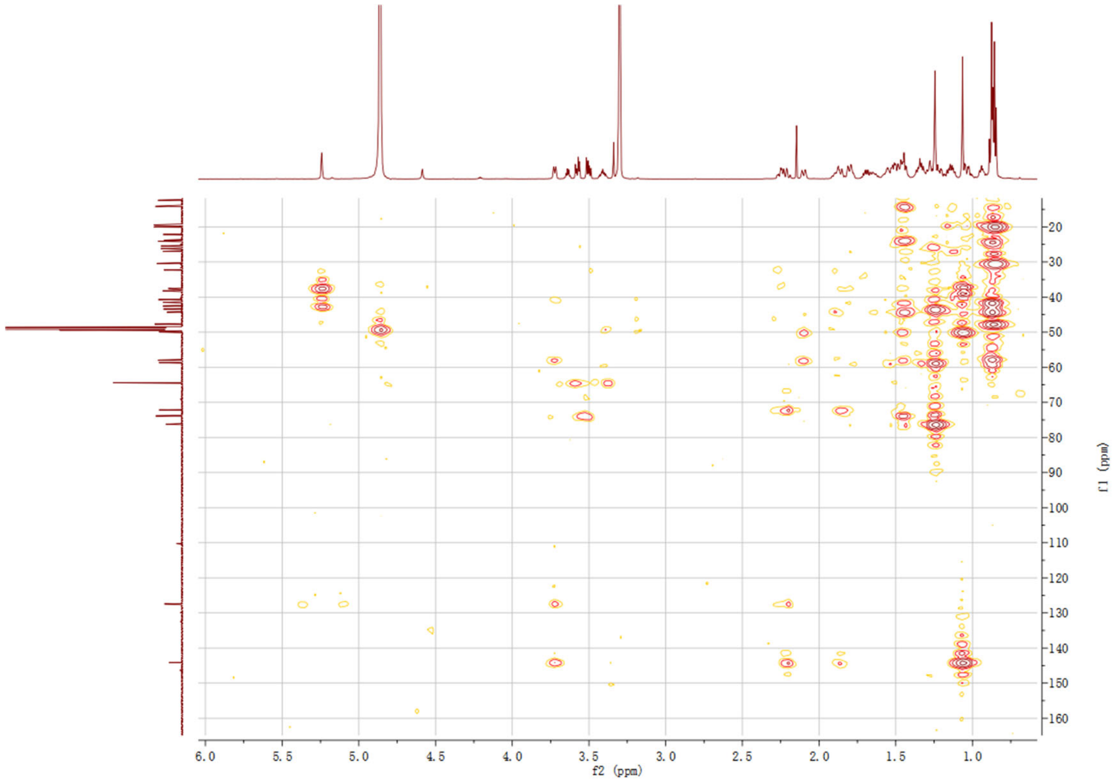

Figure S17. HMBC spectrum of compound **2** in methanol-*d*4.

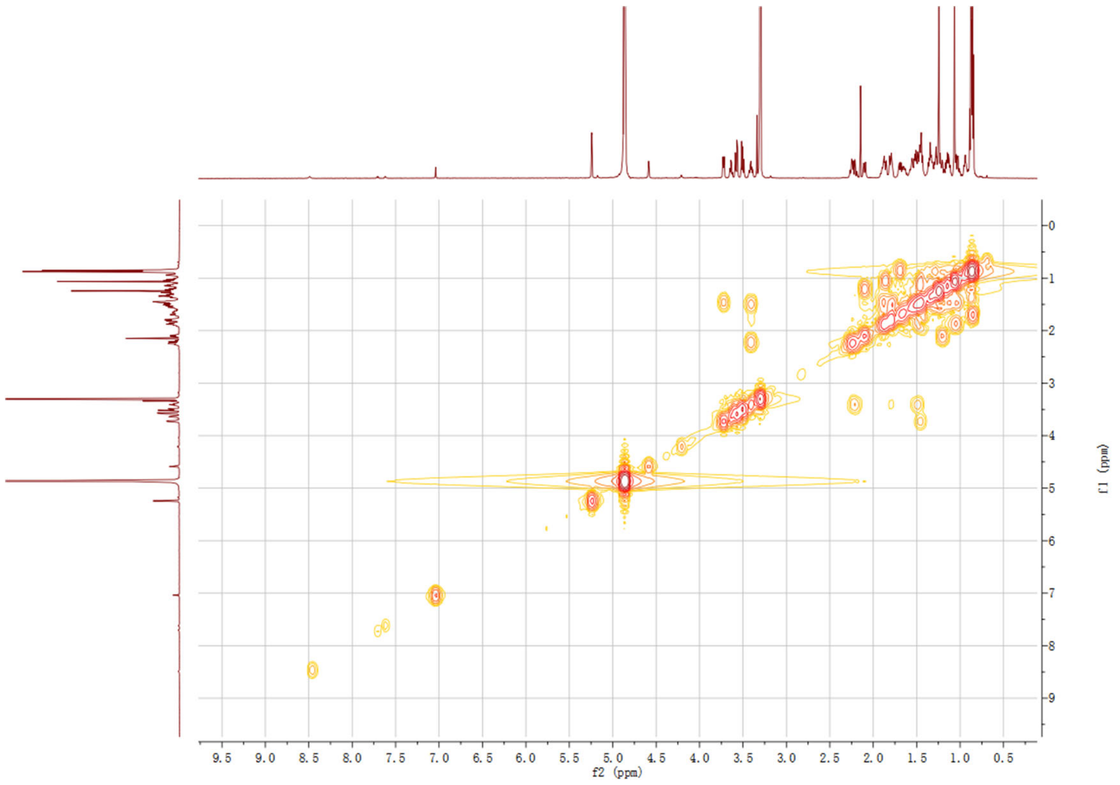

Figure S18. ^{1}H-^{1}H COSY spectrum of compound **2** in methanol-*d*4.

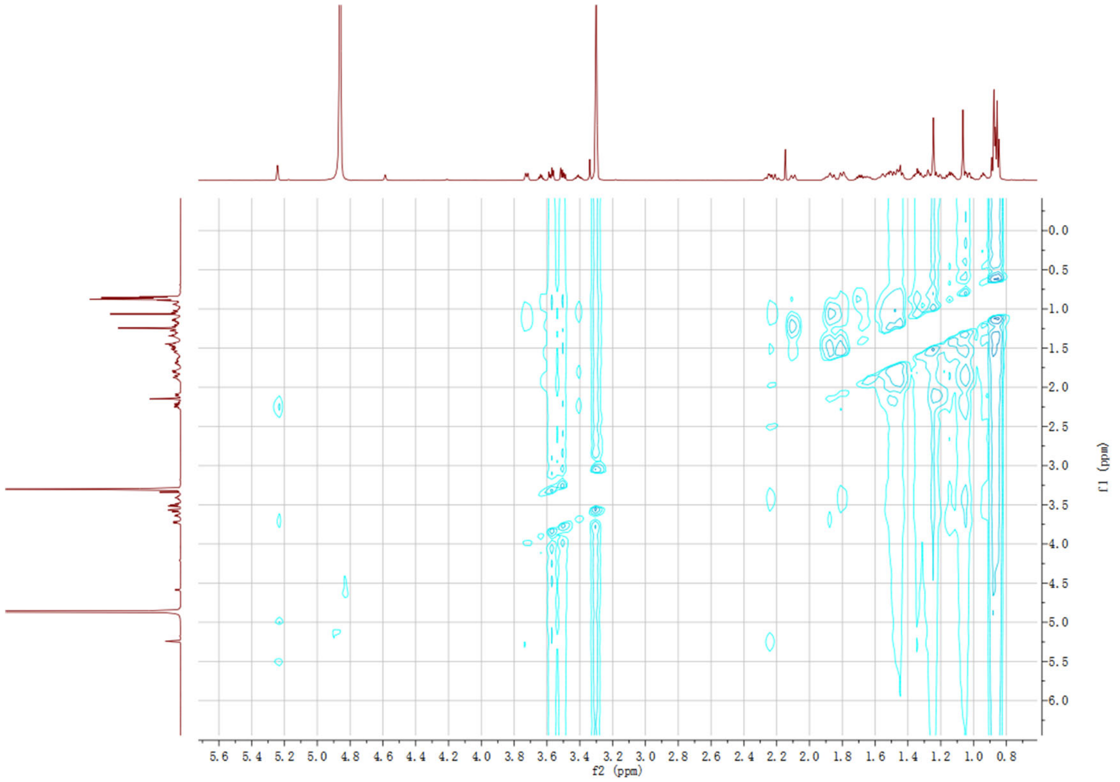

Figure S19. ROESY spectrum of compound **2** in methanol-*d*4.

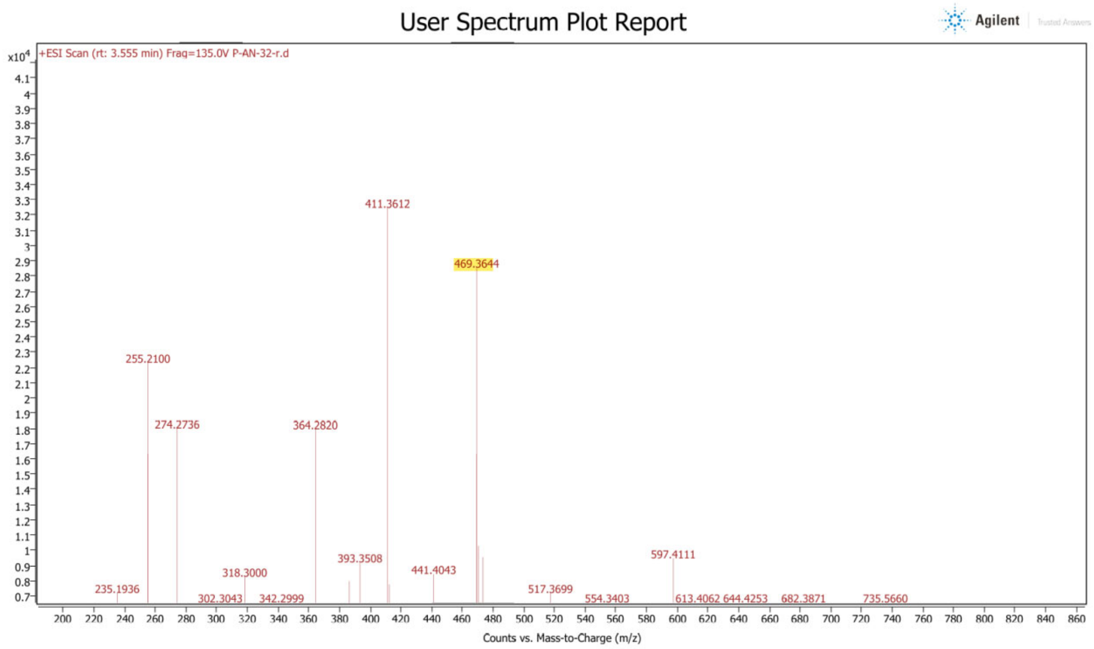

Figure S20. HR-ESI-MS spectrum of compound **2**.

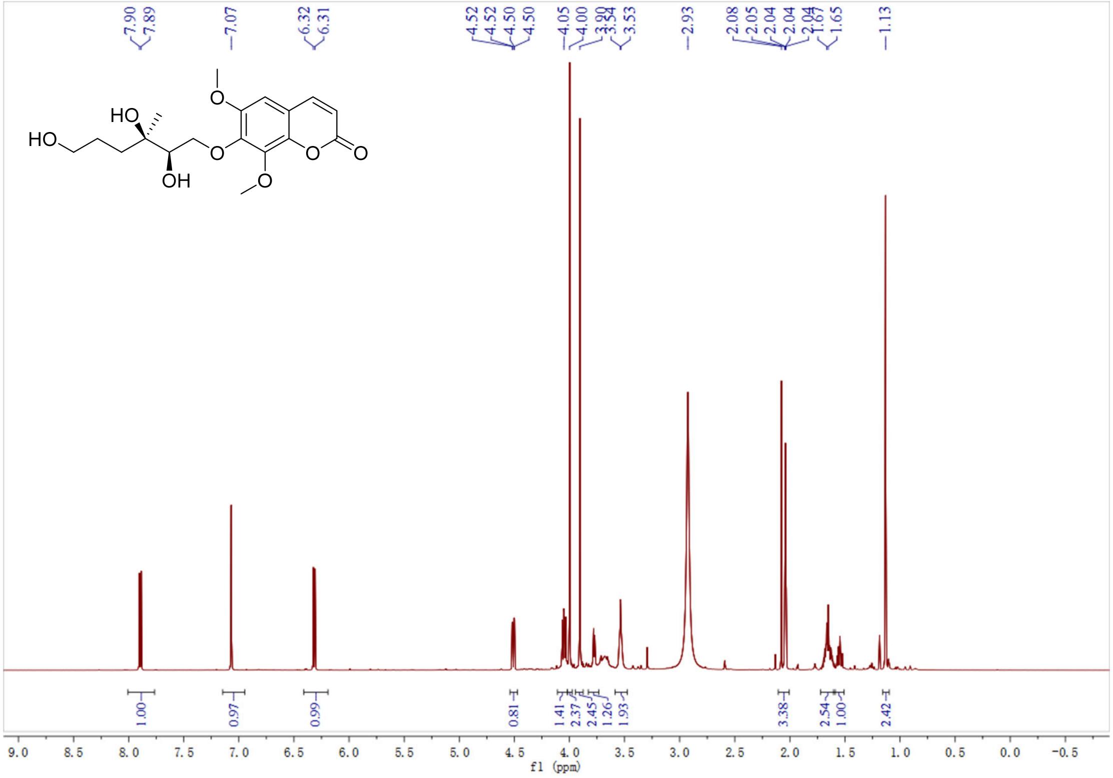

Figure S21. ^{1}H NMR spectrum of compound **3** in acetone-d_6.

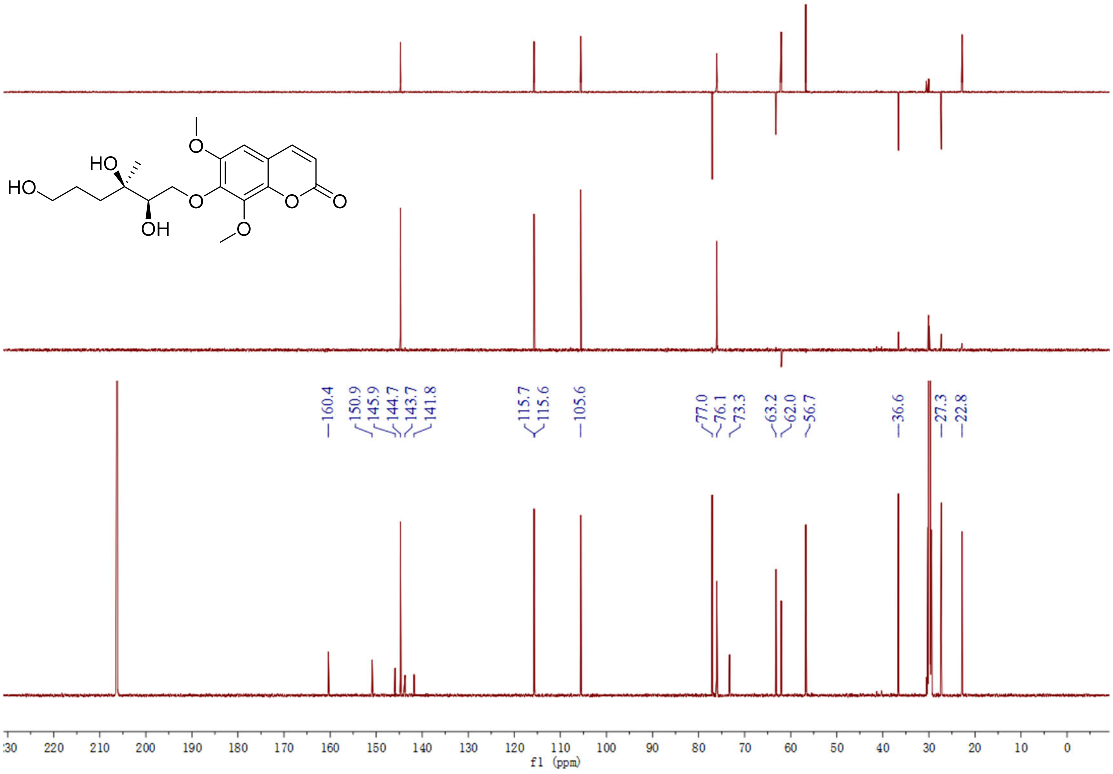

Figure S22. ^{13}C NMR and DEPT spectra of compound **3** in acetone-d_6.

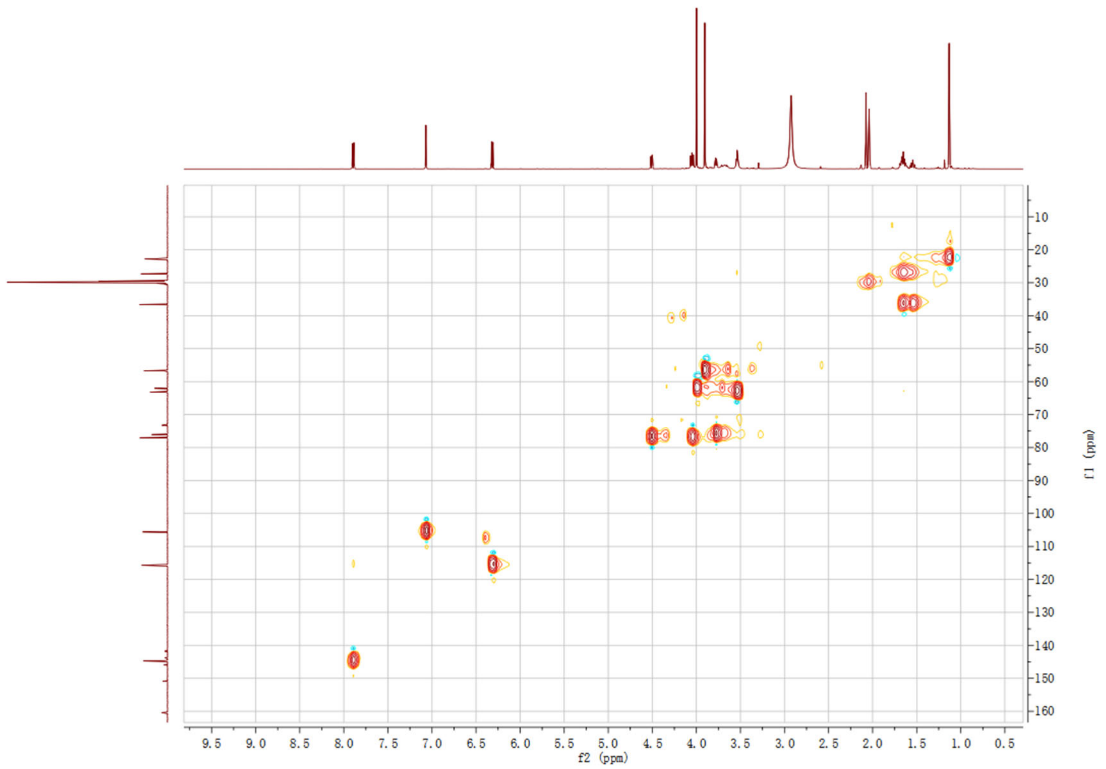

Figure S23. HSQC spectrum of compound **3** in acetone-d_6.

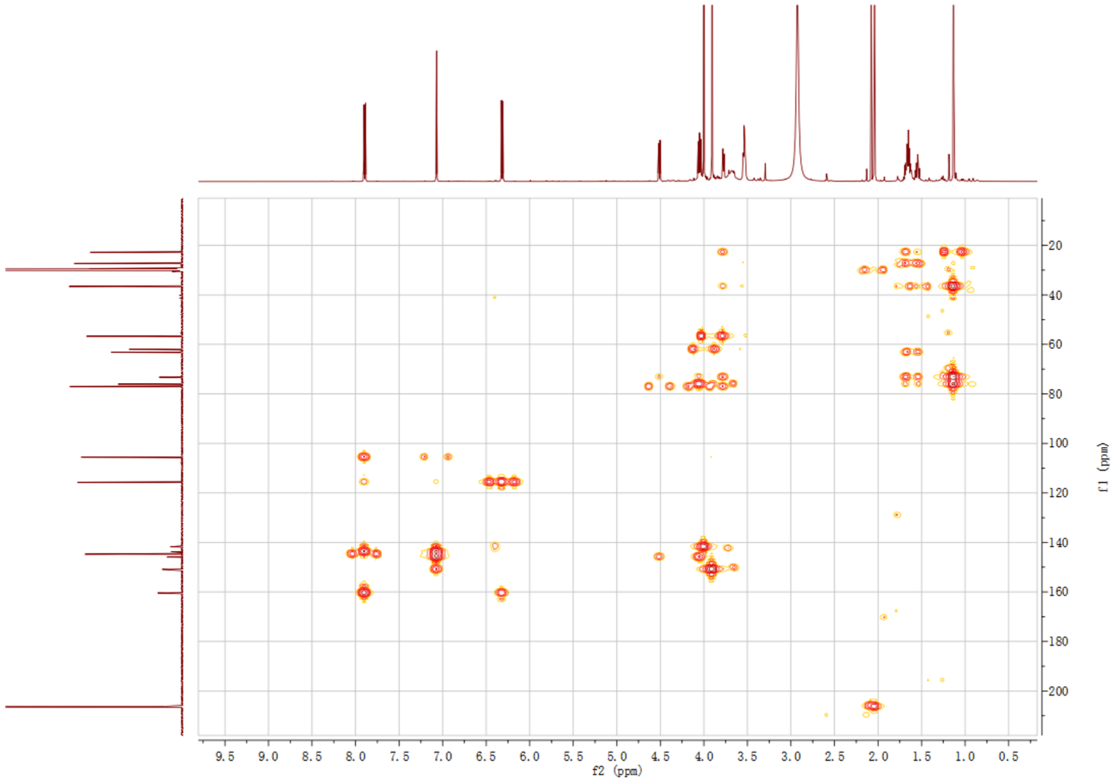

Figure S24. HMBC spectrum of compound **3** in acetone-d_6.

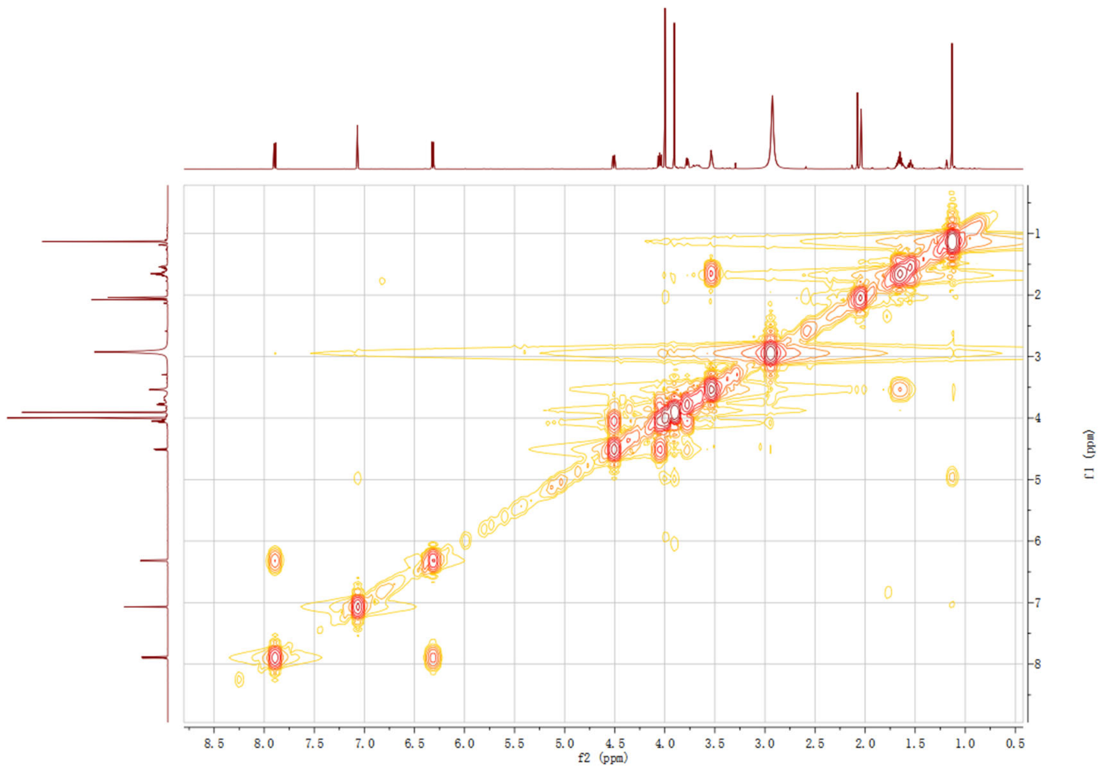

Figure S25. ^{1}H-^{1}H COSY spectrum of compound **3** in acetone-d_6.

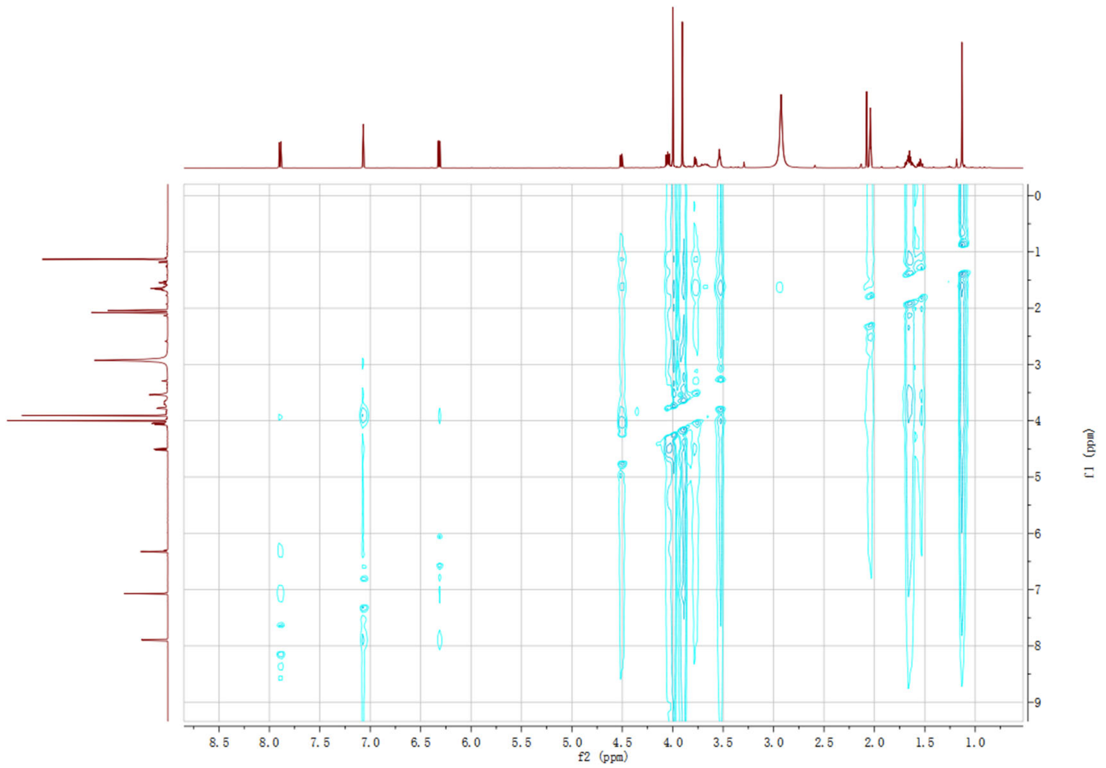

Figure S26. ROESY spectrum of compound **3** in acetone-d_6.

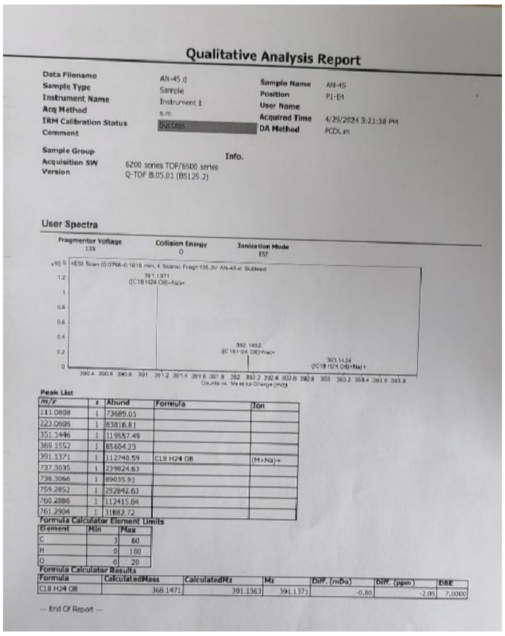

Qualitative Analysis Report

**Data Filename**	AN-45.d	**Sample Name**	AN-45
**Sample Type**	Sample	**Position**	P1-E4
**Instrument Name**	Instrument 1	**User Name**	
**Acq Method**	s.m	**Acquired Time**	4/29/2024 3:21:38 PM
**IRM Calibration Status**	Success	**DA Method**	PCDL.m
**Comment**			

Sample Group

Info.

Acquisition SW Version 6200 series TOF/6500 series Q-TOF B.05.01 (B5125.2)

User Spectra

Fragmentor Voltage	Collision Energy	Ionization Mode
135	0	ESI

Peak List

m/z	z	Abund	Formula	Ion
111.0808	1	73689.05		
223.0606	1	83816.81		
351.1446	1	119557.49		
369.1552	1	85604.23		
391.1371	1	112740.59	C18 H24 O8	(M+Na)+
737.3035	1	239824.63		
738.3066	1	89035.91		
759.2852	1	292842.63		
760.2886	1	112415.84		
761.2904	1	31882.72		

Formula Calculator Element Limits

Element	Min	Max
C	3	60
H	0	100
O	0	20

Formula Calculator Results

Formula	CalculatedMass	CalculatedMz	Mz	Diff. (mDa)	Diff. (ppm)	DBE
C18 H24 O8	368.1471	391.1363	391.1371	-0.80	-2.05	7.0000

--- End Of Report ---

Figure S27. HR-ESI-MS spectrum of compound **3**.

Rudolph Research Analytical

This sample was measured on an Autopol VI, Serial #91058
Manufactured by Rudolph Research Analytical, Hackettstown, NJ, USA.

Measurement Date : Tuesday, 04-JUN-2024

Set Temperature : 25.0

Time Delay : Disabled

Delay between Measurement : Disabled

n	Average	Std.Dev.	% RSD	Maximum	Minimum
5	9.43	0.21	2.22	9.52	9.05

S.No	Sample ID	Time	Result	Scale	OR °Arc	WLG.nm	Lg.mm	Conc.g/100ml	Temp.
1	AN-45	10:55:08 AM	9.52	SR	0.020	589	100.00	0.210	25.0
2	AN-45	10:55:14 AM	9.52	SR	0.020	589	100.00	0.210	25.0
3	AN-45	10:55:20 AM	9.52	SR	0.020	589	100.00	0.210	25.0
4	AN-45	10:55:27 AM	9.52	SR	0.020	589	100.00	0.210	25.0
5	AN-45	10:55:33 AM	9.05	SR	0.019	589	100.00	0.210	25.0

Figure S28. Optical rotation data of compound **3**.

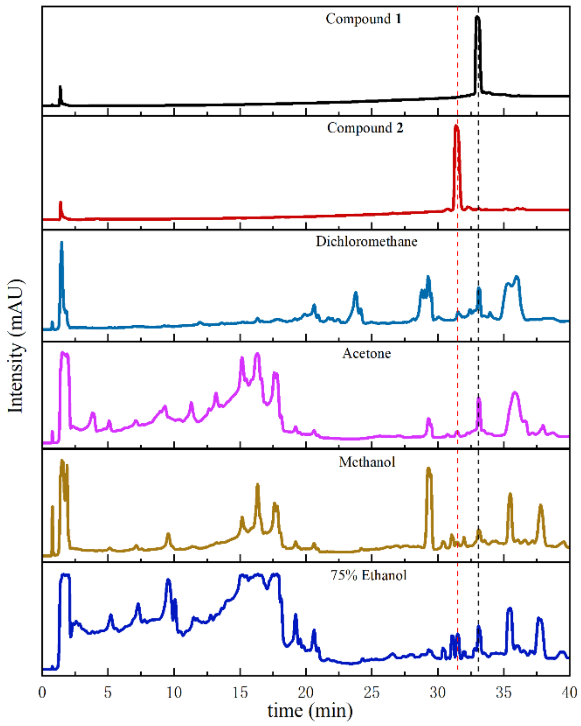

Figure S29. Qualitative and quantitative analysis of compounds **1** and **2** in *A. altissima* leaves

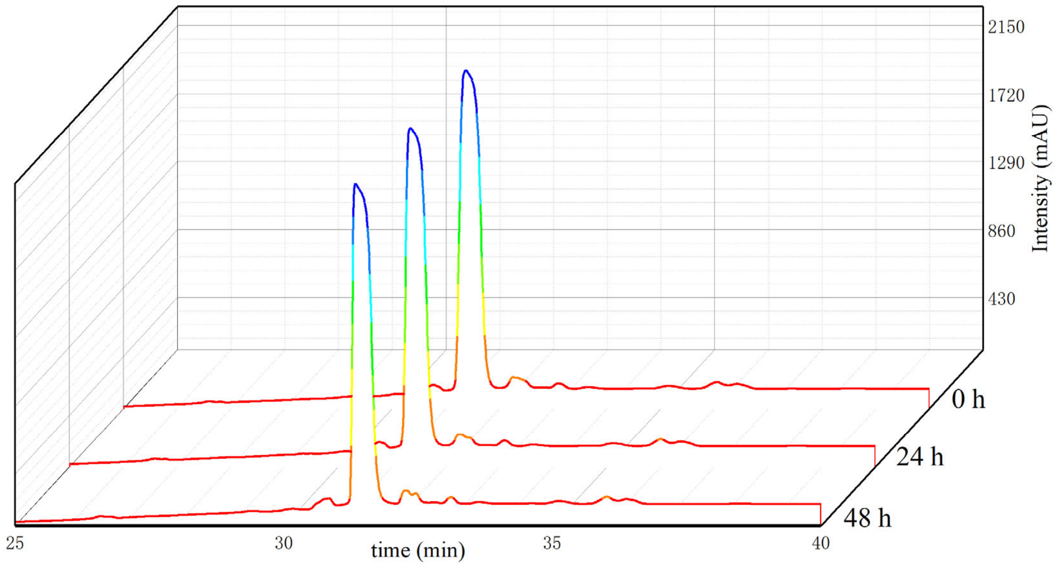

Figure S30. HPLC analysis chromatograms of compound **2** in methanol at 0, 24, and 48 hours.

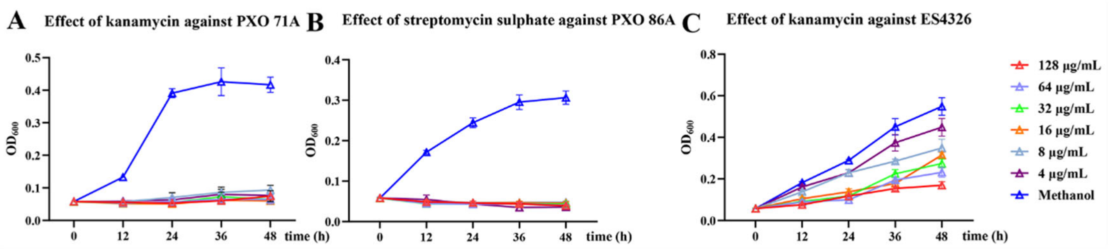

Figure S31. Growth inhibitory activity of kanamycin and streptomycin against invasive agricultural bacteria.
